# A mutation in the endonuclease domain of mouse MLH3 reveals novel roles for MutLγ during crossover formation in meiotic prophase I

**DOI:** 10.1371/journal.pgen.1008177

**Published:** 2019-06-06

**Authors:** Melissa Toledo, Xianfei Sun, Miguel A. Brieño-Enríquez, Vandana Raghavan, Stephen Gray, Jeffrey Pea, Carolyn R. Milano, Anita Venkatesh, Lekha Patel, Peter L. Borst, Eric Alani, Paula E. Cohen

**Affiliations:** 1 Department of Biomedical Sciences, Cornell University, Ithaca, NY, United States of America; 2 The Center for Reproductive Genomics, Cornell University, Ithaca, NY, United States of America; 3 Department of Molecular Biology and Genetics, Cornell University, Ithaca, NY, United States of America; Stanford University School of Medicine, UNITED STATES

## Abstract

During meiotic prophase I, double-strand breaks (DSBs) initiate homologous recombination leading to non-crossovers (NCOs) and crossovers (COs). In mouse, 10% of DSBs are designated to become COs, primarily through a pathway dependent on the MLH1-MLH3 heterodimer (MutLγ). Mlh3 contains an endonuclease domain that is critical for resolving COs in yeast. We generated a mouse (*Mlh3*^*DN/DN*^) harboring a mutation within this conserved domain that is predicted to generate a protein that is catalytically inert. *Mlh3*^*DN/DN*^ males, like fully null *Mlh3*^*-/-*^ males, have no spermatozoa and are infertile, yet spermatocytes have grossly normal DSBs and synapsis events in early prophase I. Unlike *Mlh3*^*-/-*^ males, mutation of the endonuclease domain within MLH3 permits normal loading and frequency of MutLγ in pachynema. However, key DSB repair factors (RAD51) and mediators of CO pathway choice (BLM helicase) persist into pachynema in *Mlh3*^*DN/DN*^ males, indicating a temporal delay in repair events and revealing a mechanism by which alternative DSB repair pathways may be selected. While *Mlh3*^*DN/DN*^ spermatocytes retain only 22% of wildtype chiasmata counts, this frequency is greater than observed in *Mlh3*^*-/-*^ males (10%), suggesting that the allele may permit partial endonuclease activity, or that other pathways can generate COs from these MutLγ-defined repair intermediates in *Mlh3*^*DN/DN*^ males. Double mutant mice homozygous for the *Mlh3*^*DN/DN*^ and *Mus81*^*-/-*^ mutations show losses in chiasmata close to those observed in *Mlh3*^*-/-*^ males, indicating that the MUS81-EME1-regulated crossover pathway can only partially account for the increased residual chiasmata in *Mlh3*^*DN/DN*^ spermatocytes. Our data demonstrate that mouse spermatocytes bearing the MLH1-MLH3^DN/DN^ complex display the proper loading of factors essential for CO resolution (MutSγ, CDK2, HEI10, MutLγ). Despite these functions, mice bearing the *Mlh3*^*DN/DN*^ allele show defects in the repair of meiotic recombination intermediates and a loss of most chiasmata.

## Author summary

Meiosis is a specialized cell division whereby a diploid cell undergoes one round of DNA replication followed by two rounds of division, yielding up to four haploid gametes. This process depends on tethering of maternal and paternal homologous chromosomes, and by the formation of crossovers (COs) between homologs during prophase I. COs arise from programmed double-strand breaks (DSBs), and can form via one of at least two mechanisms (class I, class II). In mouse, class I represents the major CO pathway, with the MLH1-MLH3 (MutLγ) complex being critical. MLH3 contains a conserved metal binding motif, DQHA(X)_2_E(X)_4_E, required for its endonuclease function, and this activity is postulated to represent a “resolvase”activity for class I COs. We generated a point mutant (*Mlh3*^*DN*^) in the endonuclease domain without altering the overall structure of MutLγ. *Mlh3*^*DN/DN*^ males have no spermatozoa and are infertile, yet spermatocytes have grossly normal DSBs and chromosome pairing. The *MLH3*^*DN*^ mutation permits normal loading of MutLγ, but key DSB repair factors persist in *Mlh3*^*DN/DN*^ males, indicating a temporal delay in repair and suggesting a mechanism by which alternative DSB repair pathways may be selected. Thus, the endonuclease domain of MLH3 is important for normal processing of DSB repair intermediates.

## Introduction

Meiosis is a specialized cell division process in which a diploid parental cell undergoes one round of DNA replication followed by two rounds of division, resulting in up to four haploid gametes. Successful halving of the genome during meiosis I depends on the tethering of maternal and paternal homologous chromosomes during meiotic prophase I, and their subsequent release at the first meiotic division. This tethering is ensured by homologous recombination, leading to the formation of crossovers; by synapsis, the formation of a tripartite proteinaceous structure, the synaptonemal complex, or SC between homologous chromosomes; and by cohesion between replicated sister chromatids that ensures appropriate tension on the metaphase I spindle [1,2]. Thus, recombination and synapsis are hallmarks of prophase I, and are both essential for ensuring homolog interactions leading to the formation of at least one crossover event per chromosome pair. Moreover, the correct placement, frequency, and distribution of crossovers is critical for ensuring appropriate disjunction at metaphase I and for maintaining genomic stability [1,3].

Meiotic recombination begins with the introduction of a large number of programmed double-strand breaks (DSBs), which are repaired as non-crossovers (NCOs) or crossovers (COs). Evidence for distinct NCO versus CO pathways was obtained in *S*. *cerevisiae*, where it was shown that the former occur earlier in meiotic prophase I, and subsequent work suggested that they appeared primarily through synthesis-dependent strand annealing (SDSA)[4,5]. In *M*. *musculus*, only 10% of DSBs are repaired as COs, while the majority are mostly repaired as NCOs, presumably via SDSA or other pathways, [6–9].

COs can form via one of at least two distinct mechanisms (referred to as class I and class II), each of which is used in varying degrees in different eukaryotic organisms [6,10,11]. The class I CO pathway is also known as the ZMM pathway, named after the major genes discovered in yeast that regulate this mechanism [12–18]. Class II COs, on the other hand, do not involve the ZMM proteins, but instead appear to rely on the structure-specific endonuclease (SSN), MUS81/EME1 (Mus81/Mms4 in *S*. *cerevisiae*) [6,10,11]. Class I COs also differ from class II COs in that the former are regulated by interference, the process by which placement of one CO prevents the nearby localization of a second CO, thus resulting in CO events spaced further apart than expected by chance [19].

In the class I CO pathway, DSBs are processed and resected to form single-end invasion (SEI) intermediates. This is followed by displacement of single strand DNA (ssDNA) from the recipient homolog to produce a double Holliday junction (dHJ). The yeast ZMM proteins Msh4 and Msh5 form a complex known as MutSγ that associates with a subset of these intermediate structures [20–22]. At least in yeast, this recruitment may be dependent on the STR complex, consisting of Sgs1 (BLM in mammals), Top3 and Rmi1 [23,24]. STR is proposed to act by disassembling the early recombination intermediates that would otherwise be processed through SSN-directed recombination pathways, thereby promoting either early NCO formation via SDSA, or CO formation through the capture of these recombination intermediates by the ZMM proteins, including MutSγ [23]. MutSγ is then thought to stabilize the dHJs, leading to the recruitment of a second MMR complex, MutLγ, consisting of the MutL homologs, Mlh1 and Mlh3 [25,26]. The mouse MutSγ complex associates with chromosome cores in zygonema [27], recruiting the MutLγ complex in pachynema. However, MutLγ associates with only a subset of MutSγ sites (~24–26 and 150 foci/nucleus, respectively), designating these events as class I COs [28,29].

Though not formally considered to be ZMM proteins, MLH1 and MLH3 are critical for most, if not all, class I CO events in numerous organisms [26,29–36]. In fact, the *M*. *musculus* MLH1-MLH3 heterodimer localizes to sites that are destined to become class I COs and the absence of either subunit in male spermatocytes leads to a dramatic decrease, but not complete absence, of chiasmata (the physical manifestation of a CO) [28,29,37–40]. While MutLγ is known to be recruited to sites that are preloaded with MutSγ, recent studies have shown that *S*. *cerevisiae* MutLγ can bind to single and double-stranded DNA (ssDNA, dsDNA), as well as a variety of branched DNA structures [33,41–43]. How such binding properties relate to the *in vivo* functions of MutLγ remains unclear.

Class I CO formation in *M*. *musculus* is dependent on MLH3, and on its heterodimeric interaction with MLH1 [8,29,37]. Interestingly, MLH3 recruitment precedes that of MLH1 [28]. Further analysis of MutLγ has shown that MLH3 contains a conserved metal binding motif, DQHA(X)_2_E(X)_4_E, originally discovered in the human MutL homolog, PMS2, and found to be required for human MutLα (hMLH1/hPMS2) endonuclease function [44]. This putative endonuclease motif is highly conserved in eukaryotic homologs of human PMS2 and MLH3, but not in homologs of human MLH1 and PMS1. The expectation for MLH3 is that this endonuclease function might represent a “resolvase”activity for class I COs. Studies in *S*. *cerevisiae* have shown that a single point mutation in the endonuclease motif of yeast Mlh3 (*mlh3-D523N*) disrupts its endonucleolytic activity and results in meiotic crossover defects similar to full *mlh3* (*mlh3Δ*) null mutants, yet does not affect the protein stability of Mlh3 or its interaction with Mlh1 [32]. Further analysis of the entire endonuclease domain in *S*. *cerevisiae* revealed that mutation of any conserved residue results in a null or near-null phenotype with respect to crossing over [34]. Biochemical analysis reveals that the Mlh1-mlh3D523N protein lacks the ability to nick closed circular double stranded DNA, indicating loss of endonuclease activity [33,43]. Collectively, these studies in *S*. *cerevisiae* suggest that MutLγ plays a direct role in resolving dHJs to generate COs through its endonuclease activity.

To investigate the function of the putative endonuclease domain of MLH3 in mammalian meiotic recombination, we generated a point mutant mouse (termed *Mlh3*^*DN*^) in which the endonuclease domain was disrupted at the orthologous residue to the D523N mutation in yeast, allowing the overall structure of MLH3 to remain intact, as determined by the ability to form a stable complex with MLH1. By mutating the catalytic domain of MLH3, we hypothesized that the mutant MutLγ complex would remain structurally intact and thus might reveal a functional interplay with other meiotic CO functions. We demonstrate that normal function of the MLH3 endonuclease domain is required for resolution of DSB repair intermediates towards CO formation and thus for late meiotic recombination events. *Mlh3*^*DN/DN*^ spermatocytes exhibit grossly normal DSB formation and early processing events, and normal timing of synapsis through early prophase I. *Mlh3*^*DN/DN*^ spermatocytes exhibit appropriate localization of MLH3 and MLH1 to the synaptonemal complex during pachynema, along with pro-crossover factors HEI10 and CDK2, phenotypes that are clearly different from that observed in *Mlh3*^*-/-*^ males. However, *Mlh3*^*DN/DN*^ diakinesis-staged spermatocytes show significantly fewer chiasmata compared to wild-type mice (WT), but significantly more when compared to *Mlh3*^*-/-*^ males, suggesting either that the MLH3^DN^ protein retains partial endonuclease activity, or that the presence of the MutLγ complex, albeit altered in its endonuclease capacity, can invoke MLH3-independent repair pathways to become active by interfering with normal resolution of recombination intermediates. In line with these suggestions, we find that the RecQ helicase, BLM, is upregulated throughout prophase I in *Mlh3*^*DN/DN*^ spermatocytes, perhaps aiding the recruitment of other repair proteins. To explore the increase in residual chiasmata observed at diakinesis in *Mlh3*^*DN/DN*^ males relative to that of *Mlh3*^*-/-*^ males, we demonstrate that co-incident loss of the class II CO pathway in *Mlh3*^*DN/DN*^*Mus81*^*-/-*^ double mutant males results in altered distribution of MutLγ, with an increased proportion of synapsed autosomes bearing no MutLγ foci. Furthermore, the proportion of chiasmata remaining in these double mutants is between that of *Mlh3*^*DN/DN*^ and *Mlh3*^*-/-*^ males, suggesting that MUS81-EME1 may account for only a proportion of these additional chiasmata, the mutant MutLγ retains residual resolvase activity, and/or mutant MutLγ can recruit other proteins to perform this resolvase activity at a subset of recombination intermediate sites. Collectively, our data show that the endonuclease activity of MLH3 is important for normal processing of DSB repair intermediates through the Class I pathway.

## Results

### *Mlh3*^*DN/DN*^ males are infertile

To investigate the meiotic requirement for the presence of a functional endonuclease domain in mammalian MLH3, we generated a mouse line with a point mutation in a conserved endonuclease motif located in the *M*. *musculus* protein: DQHAAHERIRLE [44,45]. Specifically, we replaced the aspartic acid "D" in amino acid position 1185, with an asparagine "N" by changing GAC to AAC in the genomic sequence, termed MLH3^DN^ throughout. Extrapolating from an analogous mutation in the *S*. *cerevisiae* gene, this D-to-N replacement is predicted to disrupt the endonuclease function of MLH3 while maintaining its ability to interact with MLH1 ([32] S1 Fig). Mice were maintained on a C57Bl/6J background throughout the study.

Male *Mlh3*^*+/DN*^ mice were phenotypically similar to WT littermates and displayed full fertility. *Mlh3*^*DN/DN*^ males are also grossly normal when compared to WT littermates, survive into adulthood, and live normal lifespans. *Mlh3*^*DN/DN*^ males also exhibit normal mating behaviors as determined by observing a vaginal plug in WT females the morning after mating. However, breeding between multiple sets of *Mlh3*^*DN/DN*^ males and WT females never resulted in offspring over a four-year period.

**Fig 1:**
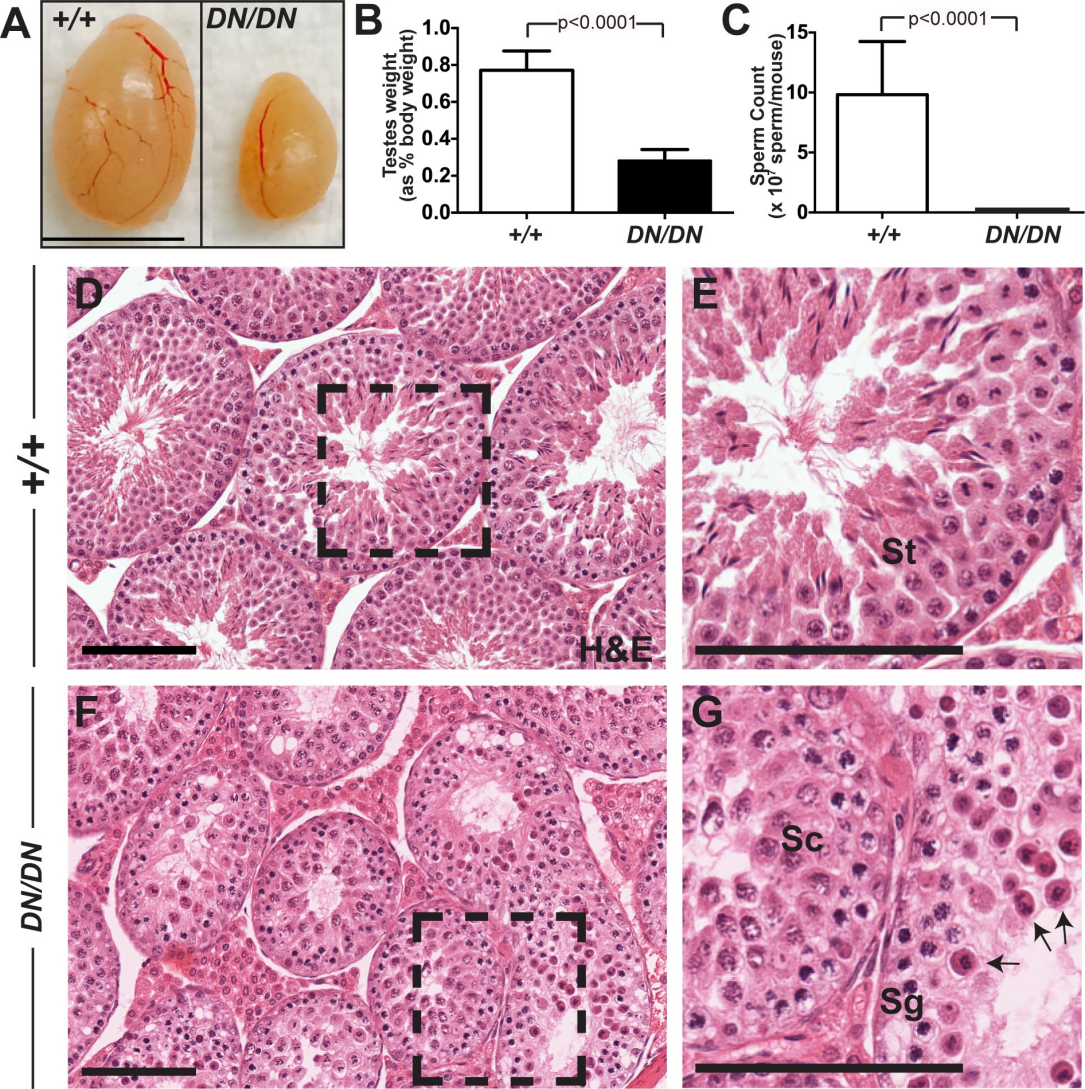
*Mlh3*^*DN/DN*^ males show a sterile phenotype. (A, B) *Mlh3*^*DN/DN*^ adult male testes are significantly smaller when compared to WT littermates (*Mlh3*^*DN/DN*^—0.28% of total body weight ± 0.06 standard deviation (s.d.), n = 13; WT—0.77% ± 0.1, n = 15; p < 0.0001, unpaired t-test with Welch's correction) and (C) have zero sperm in the epididymis (WT—9.8 x 10^7^ ± 4.4 sperm/mouse; p < 0.0001; unpaired t-test with Welch's correction, n = 10 and 12 mice, respectively; error bars show standard deviation). Hemotoxylin and eosin stained (D, E) WT testes show the presence of meiotic and post-meiotic cells whereas (F, G) *Mlh3*^*DN/DN*^ are absent of spermatids and spermatozoa. Higher magnification of WT and *Mlh3*^*DN/DN*^ testes sections are shown in E and G, respectively. Black arrows in (G) indicate metaphase I spermatocytes in the *Mlh3*^*DN/DN*^ seminiferous tubule lumen. Sg, spermatogonia; Sc, prophase I spermatocytes; St, postmeiotic spermatids. Scale bar is 100 μm. Images were taken at 40X magnification.

Similar to the situation seen for *Mlh3*^*-/-*^ males [29], *Mlh3*^*DN/DN*^ males show complete infertility, accompanied by significantly reduced testes size when compared to WT (Fig 1A and 1B; p < 0.0001) and the absence of spermatozoa in the epididymides (Fig 1C; p < 0.0001). Whereas histological cross-sections of testes stained with hemotoxylin and eosin from WT males showed the presence of meiotic and post-meiotic cells within the seminiferous epithelium, testis sections from *Mlh3*^*DN/DN*^ males were devoid of spermatids, but showed the presence of spermatogonia and spermatocytes (Fig 1D–1G). In addition, metaphase I spermatocytes were observed in the tubular lumen of *Mlh3*^*DN/DN*^ mice (Fig 1G, black arrows). Thus, mutation of the endonuclease domain of *Mlh3* in the mouse results in a sterility phenotype grossly similar to that seen in *Mlh3*^*-/-*^ mice.

### *Mlh3*^*DN/DN*^ spermatocytes, like those of *Mlh3*^*-/-*^ mice, exhibit grossly normal DSB formation and synapsis

To investigate the progression of meiotic recombination, prophase I chromosome spreads were prepared from WT, *Mlh3*^*DN/DN*^, and *Mlh3*^*-/-*^ adult males and stained for a variety of markers involved in synapsis and recombination. Chromosome spreads were stained with antibodies against γH2AX, the phosphorylated form of histone H2AX, as a marker of DSBs [46,47]. In spermatocyte preparations from WT males, γH2AX signal is abundant throughout the nucleus at leptonema, coincident with the induction of several hundred DSBs [1,47]. The γH2AX signal declines in zygonema as DSBs are processed for repair [47,48]. In pachynema and diplonema, γH2AX signal is absent from the autosomes, but emerges throughout the sex body due to meiotic sex chromosome inactivation (MSCI) ([49]; S2A–S2D Fig). Spermatocytes from both *Mlh3*^*DN/DN*^ and *Mlh3*^*-/-*^ males exhibit the same γH2AX signal and temporal dynamics as observed in WT spermatocytes, with abundant staining in leptonema, slightly reduced signaling in zygonema, followed by the absence of γH2AX signal on the autosomes of pachytene and diplotene spermatocytes, except at the sex body (S2F–S2I and S2K–S2N Fig). We do not see specific persistent γH2AX signal on the autosomes at pachynema in *Mlh3*^*-/-*^ spermatocytes [50], unless we markedly increase our imaging exposure time γH2AX (S2E, S2J and S2O Fig; white arrows). Under these conditions, we see persistent foci of γH2AX in spermatocytes from WT and from *Mlh3*^*DN/DN*^ spermatocytes also. Thus, in our hands, we see no specific persistence in autosomal γH2AX signal through pachynema in mice lacking MLH3 or harboring a mutation within the endonuclease domain of MLH3.

Spermatocyte chromosome spreads from WT and *Mlh3*^*DN/DN*^ males were stained with antibodies against synaptonemal complex (SC) components, SYCP3 and SYCP1, marking the axial/lateral elements and the transverse filaments, respectively. Prophase I progression in WT spreads is characterized by the initial accumulation of SYCP3 signal in discrete dots along chromosomes at leptonema, and these dots gradually coalesce into continuous filaments along the chromosome cores in zygonema (S2Q Fig). At this time, SYCP1 appears in patches along the SYCP3 signal, indicating that synapsis is occurring. By late zygonema, most of the chromosome core is now labeled with SYCP1, and by pachynema synapsis is complete, as demonstrated by complete overlap of the SYCP3/SYCP1 signals on the autosomes. For the sex chromosomes, synapsis only occurs at the pseudoautosomal region (PAR). After meiotic recombination occurs, the SC begins to degrade in diplonema, and the homologs are no longer tethered to one another except at CO sites (S2P–S2S Fig).

Synapsis appears normal in *Mlh3*^*DN/DN*^ spermatocytes with discrete accumulation of SYCP3 on the chromosomes in leptonema, followed by continued accumulation of SYCP3 along the chromosomes as SYCP1 appears in patches in zygonema (S2T and S2U Fig). Complete synapsis of the autosomes and the PAR is observed in pachynema with co-localization of SYCP1 and SYCP3 (S2V Fig). Desynapsis is then observed in diplonema with the degradation of the SC (S2W Fig). Thus, synapsis in *Mlh3*^*DN/DN*^ spermatocytes appears unaffected by loss of the endonuclease activity of MLH3, a result similar to that seen for complete loss of MLH3 protein.

### *Mlh3*^*DN/DN*^ spermatocytes show increased localization of the single strand DNA binding protein RPA and the strand exchange protein RAD51 through early prophase I and retention of RAD51 into pachynema

**Fig 2:**
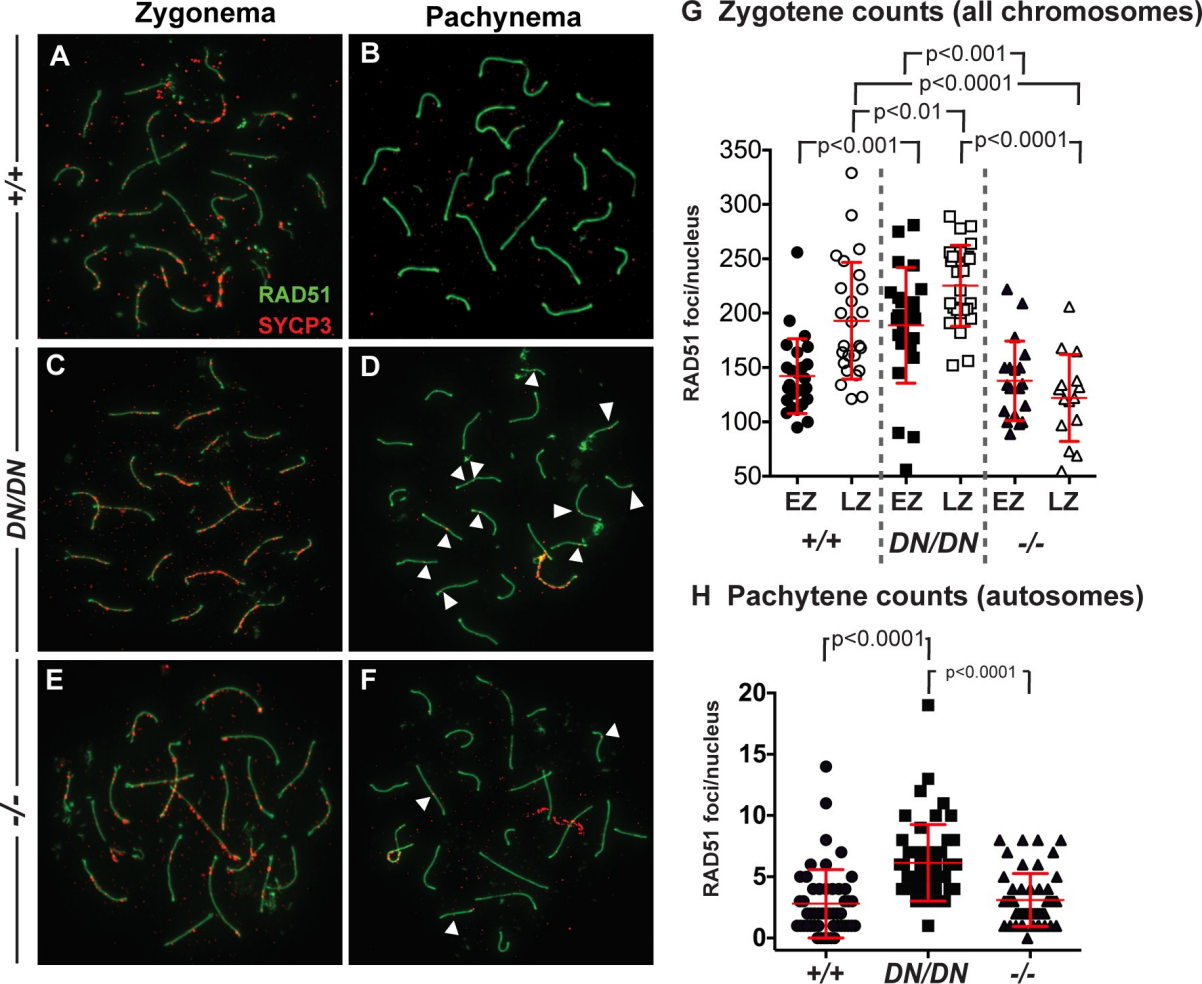
*Mlh3*^*DN/DN*^ spermatocytes show a persistence of RAD51 foci in pachynema. The localization of RAD51 (green) on synaptonemal complex protein SYCP3 (red) is observed in (A, B) WT and (C, D) *Mlh3*^*DN/DN*^ male spermatocytes in zygonema and pachynema. (A, C, E, G) In early zygonema, WT cells show high numbers of RAD51 associated with the chromosome cores while *Mlh3*^*DN/DN*^ exhibit even higher RAD51 foci (WT mean ± standard deviation = 142.1 ± 34.5 foci, *Mlh3*^*DN/DN*^ mean ± s.d. = 188.8 ± 53.3 foci; p values indicated on graph, by unpaired t-test with Welch’s correction, and using Bonferroni’s adjustment for multiple comparison). In late zygonema, *Mlh3*^*DN/DN*^ spermatocytes continue to have higher RAD51 foci than WT (WT mean ± s.d. = 193.0 ± 53.7 foci, *Mlh3*^*DN/DN*^ mean ± s.d. = 225.3 ± 37.3 foci; p values indicated on graph, by unpaired t-test with Welch’s correction, and using Bonferroni’s adjustment for multiple comparison). *Mlh3*^*-/-*^ late zygotene cells show significantly fewer RAD51 focus counts (mean = 122.1 ± 39.9 foci) when compared to WT and *Mlh3*^*DN/DN*^ (p values indicated on graph, by unpaired t-test with Welch’s correction, and using Bonferroni’s adjustment for multiple comparison). In all cases, at least 3 mice were assessed for each genotype and at least 20 cells per mouse (B, D, F, H). In pachynema, WT cells exhibit a dramatic decrease of very few to no RAD51 associated with the autosomes while *Mlh3*^*DN/DN*^ cells show persistent RAD51 (WT mean ± s.d. = 2.8 ± 2.8 foci, *Mlh3*^*DN/DN*^ mean ± s s.d. = 6.1 ± 3.1 foci; p<0.0001, unpaired t-test). *Mlh3*^*-/-*^ pachytene cells exhibit comparable RAD51 focus counts (mean ± s.d. = 3.1 ± 2.1 foci) when compared to WT (p = 0.55 by unpaired t-test), but significantly fewer than *Mlh3*^*DN/DN*^ spermatocytes (p<0.0001 by unpaired t-test). Note that sex chromosome-associated RAD51 staining was excluded from counts at pachynema. For all chromosome imaging and foci counts, at least three mice of each genotype were observed for each staining set.

Early DSB repair events were monitored by examining localization of the RecA strand exchange protein, RAD51, on chromosome cores of the autosomes throughout prophase I [51,52]. In WT mice, RAD51 localizes to chromosome cores of early and late zygotene cells as discrete foci at a high frequency (EZ and LZ, respectively; Fig 2A and 2G). Compared to WT littermates in early and late zygonema, RAD51 counts in spermatocytes from *Mlh3*^*DN/DN*^ males were significantly elevated (Fig 2C and 2G; p<0.001 and p<0.01, respectively, by unpaired t-test with Welch’s correction). However, while early zygotene RAD51 counts were indistinguishable in *Mlh3*^*-/-*^ spermatocytes compared to WT (Fig 2E and 2G), they were significantly lower than that seen at the equivalent stage in *Mlh3*^*DN/DN*^ males (p<0.001 by unpaired t-test with Welch’s correction). By late zygonema, the RAD51 counts were significantly lower in *Mlh3*^*-/-*^ spermatocytes compared to WT and *Mlh3*^*DN/DN*^ animals (p<0.001 by unpaired t-test with Welch’s correction). By pachynema, RAD51 foci frequency in spermatocytes from WT mice decreased to very low numbers, as did that of *Mlh3*^*-/-*^ males (Fig 2B, 2F and 2H; p = 0.55 unpaired t-test). In contrast, focus counts in pachytene spermatocytes from *Mlh3*^*DN/DN*^ males remained significantly elevated following the pattern first seen in zygonema (Fig 2D and 2H; p<0.0001).

The localization and accumulation of single strand DNA binding protein RPA, which associates with chromosomes from zygonema through until early pachynema, was also explored. (S3 Fig). In leptonema and zygonema, RPA focus counts on chromosome cores were significantly elevated in *Mlh3*^*DN/DN*^ animals compared to WT (p<0.01 and p<0.001, respectively, unpaired t-test with Welch’s correction), similar to the increased focus frequency observed for RAD51. However, unlike RAD51, the RPA focus counts were not significantly different between genotypes at pachynema and diplonema (S3 Fig). These observations suggest either that there is a prolonged period of DSB induction in *Mlh3*^*DN/DN*^ animals, or that there is a lag time in the turnover of DSB repair intermediates in early prophase I. Taken together with the persistent RAD51 localization, these observations suggest that, in *Mlh3*^*DN/DN*^ spermatocytes, there is a persistence of DSB repair intermediates loaded with RPA in zygonema, and that these intermediates continue to persist as they accumulate RecA homolog proteins, with RAD51 remaining on chromosome cores of *Mlh3*^*DN/DN*^ spermatocytes through pachynema. We hypothesize, based on these observations, that RPA accumulation in leptonema and RAD51 accumulation in zygonema are affected by loss or mutation of MLH3 protein, suggesting an early function for MutLγ in establishing appropriate DSB repair intermediates that is not confined to CO pathway fate [42,53]. We hypothesize that early DSB repair events occur within the normal timeframe in mice lacking MLH3 protein entirely, but at an even faster rate than in WT spermatocytes, because in late zygotene RAD51 counts in *Mlh3*^*-/-*^ mutants have declined to levels seen in pachytene. The repair of these DSBs in *Mlh3*^*-/-*^ mutants may occur through repair pathways that differ from those utilized in WT-derived spermatocytes. We hypothesize that in *Mlh3*^*DN/DN*^ mutants DSBs are not repaired efficiently, or there is an extended period of DSB induction resulting from feedback mechanisms that lead to a persistence of RPA and RAD51 foci.

### *Mlh3*^*DN/DN*^ pachytene spermatocytes show a hyper-accumulation and persistence of BLM

Bloom's syndrome mutated (BLM) is a mammalian RecQ DNA helicase whose *S*. *cerevisiae* ortholog, Sgs1, was shown to promote the resolution of complex multi-chromatid joint molecule intermediates, that may result from SEI events, into both NCOs and COs [23,24]. During prophase I in WT male spermatocytes, BLM localizes to the chromosomal cores at a high frequency in zygonema and diminishes to a few foci in pachynema [54–56]. Recently, we showed that loss of MLH3 results in up-regulated BLM localization during prophase I, along with persistence of BLM on chromosome cores through late pachynema [56].

**Fig 3:**
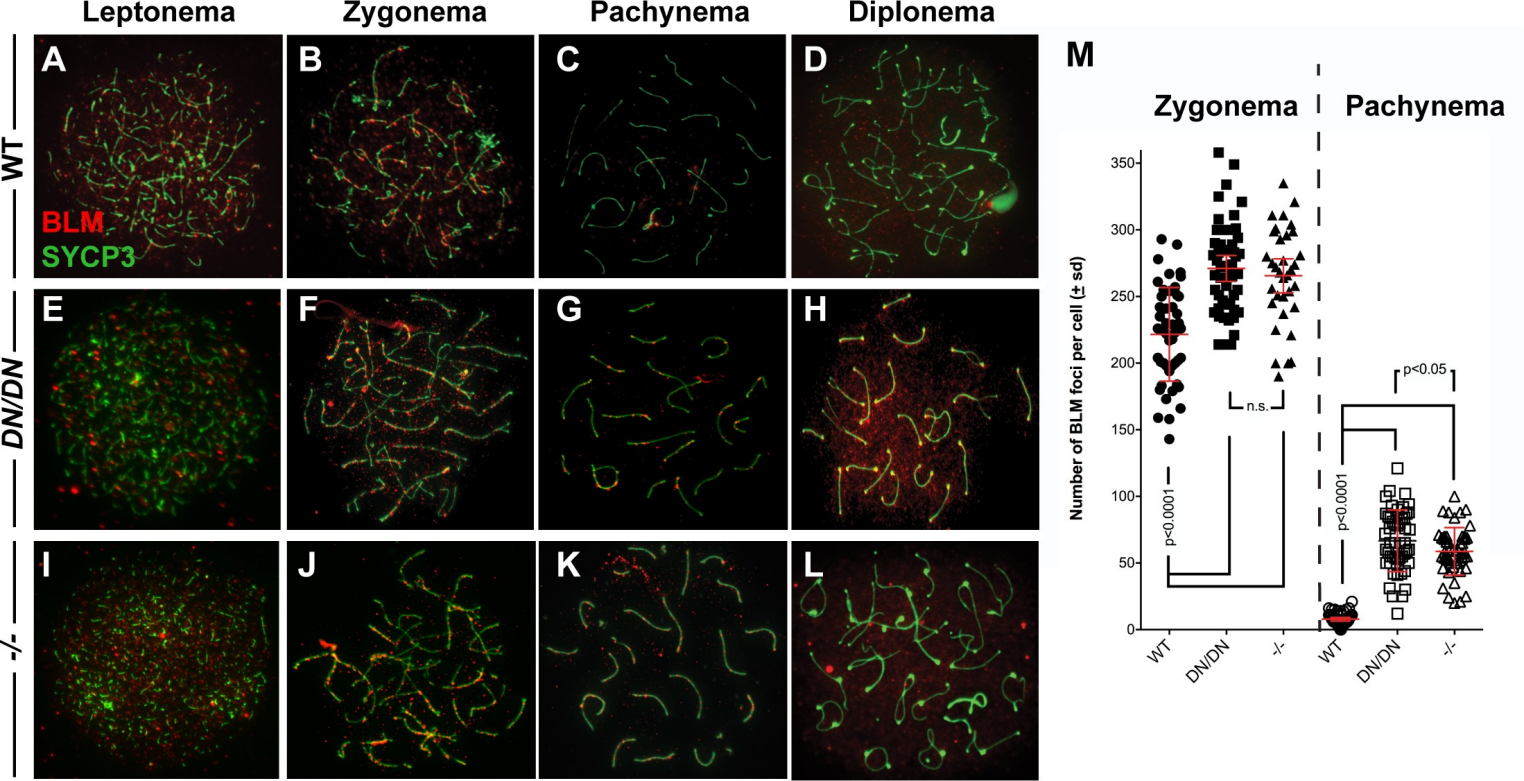
*Mlh3*^*DN/DN*^ spermatocytes show a persistence of BLM in pachynema. Meiotic spreads chromosomes from (A-D) WT, (E-H) *Mlh3*^*DN/DN*^, and (I-L) *Mlh3*^*-/-*^ males showing the localization of BLM (red) on synaptonemal complex protein SYCP3 (green) throughout the progression of prophase I. (B, F, J, M) In zygonema, *Mlh3*^*DN/DN*^ and *Mlh3*^*-/-*^ cells show significantly more BLM foci localized to the chromosome cores than WT (WT mean ± s.d. = 221.6 ± 35.1 foci, *Mlh3*^*DN/DN*^ mean ± s.d. = 271.1 ± 34.2 foci, *Mlh3*^*-/-*^ mean ± s.d. = 265.5 ± 36.6 foci; p values indicated on graph, by unpaired t-test with Welch’s correction, and using Bonferroni’s adjustment for multiple comparison). (C, D, K, M) In pachynema, BLM is no longer present on the chromosome cores of WT cells whereas *Mlh3*^*DN/DN*^ and *Mlh3*^*-/-*^ cells show hyper-accumulation of BLM on the autosomes and the sex body (WT mean ± s.d. = 7.8 ± 4.7 foci, *Mlh3*^*DN/DN*^ mean ± s.d. = 66.7 ± 23.2 foci, *Mlh3*^*-/-*^ mean ± s.d. = 58.7 ± 17.8 foci; p values indicated on graph, by unpaired t-test with Welch’s correction, and using Bonferroni’s adjustment for multiple comparison), which persists into diplonema. At zygonema, the frequency of BLM foci in *Mlh3*^*DN/DN*^ and *Mlh3*^*-/-*^ cells is not statistically different, whereas by pachynema, the number of BLM foci remains significantly elevated in *Mlh3*^*DN/DN*^ spermatocytes, relative to that observed in *Mlh3*^*-/-*^ cells (p<0.05, unpaired t-test). By diplonema, clear and quantifiable foci are no longer observed in spermatocytes from mice of all genotypes. However, the background staining intensity for these images is observed at standardize exposure settings for image acquisition, so the background staining is comparable between genotypes. For all chromosome imaging and foci counts, at least 3 mice of each genotype were observed for each staining set.

To determine if the disruption of the MLH3 endonuclease domain affects the localization of BLM in a similar fashion, to *Mlh3*^*-/-*^, we stained prophase I chromosome spreads with an antibody against BLM. In zygonema, as previously reported, WT cells show the accumulation of BLM foci on the cores in high numbers, and this frequency is elevated in spermatocytes from both *Mlh3*^*DN/DN*^ and *Mlh3*^*-/-*^ spermatocytes (Fig 3A, 3B, 3E, 3F, 3I, 3J and 3M; p<0.0001 unpaired t-test). This is similar to that reported previously for *Mlh3*^*-/-*^ spermatocytes [56]. In early to mid-pachynema, BLM localization on chromosome cores persists in a small percentage of WT spermatocytes, but the number of foci is very much reduced at this stage (Fig 3C, 3D and 3M). In contrast, all spermatocytes from *Mlh3*^*DN/DN*^ and *Mlh3*^*-/-*^ spermatocytes show persistent BLM focus localization along chromosome cores (Fig 3G, 3H, 3K and 3L) at a frequency that is elevated above that of WT spermatocytes (Fig 3M, p<0.0001 unpaired t-test). Moreover, the number of BLM foci at pachynema in *Mlh3*^*DN/DN*^ spermatocytes is significantly elevated relative to that seen in *Mlh3*^*-/-*^ spermatocytes (Fig 3M, p<0.05 unpaired t-test). By diplonema this difference appears even greater, with BLM localization in *Mlh3*^*DN/DN*^ spermatocytes persisting in stretches along the cores, and being lost entirely in *Mlh3*^*-/-*^ spermatocytes (Fig 3D, 3H and 3L). Thus, altered MLH3 endonuclease function, like complete loss of MLH3, leads to persistence of BLM helicase on chromosome cores in late prophase I, but at an elevated frequency in *Mlh3*^*DN/DN*^ spermatocytes relative to *Mlh3*^*-/-*^ spermatocytes.

### Increased localization of pro-crossover factor, RNF212, but not MSH4 in pachytene spermatocytes from *Mlh3*^*-/-*^ and *Mlh3*^*DN/DN*^ adult male mice

“Crossover designation” is defined as the process by which class I COs are selected from an excess pool of DSB repair intermediates. In mouse, the 250+ DSBs are processed through zygonema into various repair pathways, and only a subset of these will proceed towards a class I CO fate [1]. These sites become “licensed” for crossing over through the accumulation of the MutS homolog heterodimer, MutSγ (MSH4 and MSH5; [27,57]). The MutSγ complex then serves as an early pro-crossover factor by recruiting the MutLγ complex to a select subset of sites and it is these sites that will become “designated” as class I CO events. Notably, all of the 150+ MutSγ sites must be repaired, either as a CO or an NCO, which means that approximately ~125 MutSγ sites must leave the class I CO pathway and undergo repair through an alternate CO pathway or via an NCO pathway, a situation that is unlike that seen in *S*. *cerevisiae* where the number of MutSγ sites appear to correspond more closely to the number of CO events [20].

While the mechanism by which only a subset of MutSγ foci are retained through pachynema remains unclear, studies from a number of groups have implicated the Zip3-like protein, RNF212, in this process [58,59]. RNF212 has been shown to co-localize with the majority of MutSγ foci in spermatocytes from WT males and is thought to act as a pro-crossover factor by stabilizing these MutSγ-loaded events [59]. As such, the number of RNF212 foci on chromosome cores is pared down through pachynema in a similar fashion to that of MutSγ [59,60]. Moreover, in mouse mutants that disrupt this paring down process, both RNF212 and MutSγ focus counts remain elevated, but equivalent, throughout prophase I [50,61].

To investigate how loss of MLH3 endonuclease function could affect this paring down process, we analyzed RNF212 and MSH4 focus dynamics on chromosome spreads throughout prophase I from WT, *Mlh3*^*DN/DN*^, and *Mlh3*^*-/-*^ adult male mice (S4 Fig). For both RNF212 (S4A–S4M Fig) and MSH4 (S4N–S4Z Fig), we find the expected paring down of focus counts from early pachynema (EP) to late pachynema (LP) in spermatocytes from WT, *Mlh3*^*DN/DN*^, and *Mlh3*^*-/-*^ adult males. In all three cases, RNF212 and MSH4 foci appear on chromosome cores in zygonema (S4B, S4F, S4J, S4O, S4S and S4W Fig), persist at high levels in early pachynema (S4C, S4G, S4K, S4P, S4T and S4X Fig), and then are reduced to approximately 1–2 foci per chromosome in late pachynema (S4D, S4H, S4L, S4Q, S4U and S4Y Fig). Quantification of RNF212 and MSH4 focus numbers in early and late pachytene spermatocytes from WT, *Mlh3*^*DN/DN*^, and *Mlh3*^*-/-*^ adult male reveals the expected statistically significant decline in these foci through pachynema (S4M and S4Z Fig; p<0.0001 Mann-Whitney U Test for all). However, the levels of RNF212 foci in both early and late pachynema are significantly higher in spermatocytes from *Mlh3*^*DN/DN*^ and *Mlh3*^*-/-*^ adult males compared to that seen in WT spermatocytes (S4M Fig; p<0.001 Mann-Whitney U test for all). Thus, while the dynamics of RNF212 (high in early and low in late pachynema) are evident in *Mlh3*^*DN/DN*^ and *Mlh3*^*-/-*^ adult males, their focus counts at each of these stages are significantly elevated compared to equivalently-staged WT RNF212 counts. By contrast, at both early and late pachynema, MSH4 counts did not differ between spermatocytes from WT, *Mlh3*^*DN/DN*^ and *Mlh3*^*-/-*^ adult males at both early and late pachynema (S4Z Fig). Thus, mice bearing no MLH3 or catalytically defective MLH3 show a phenotypic divergence in RNF212 and MSH4 focus counts in pachytene spermatocytes compared to WT.

### *Mlh3*^*DN/DN*^ pachytene staged spermatocytes exhibit normal localization of MLH3 and MLH1

**Fig 4:**
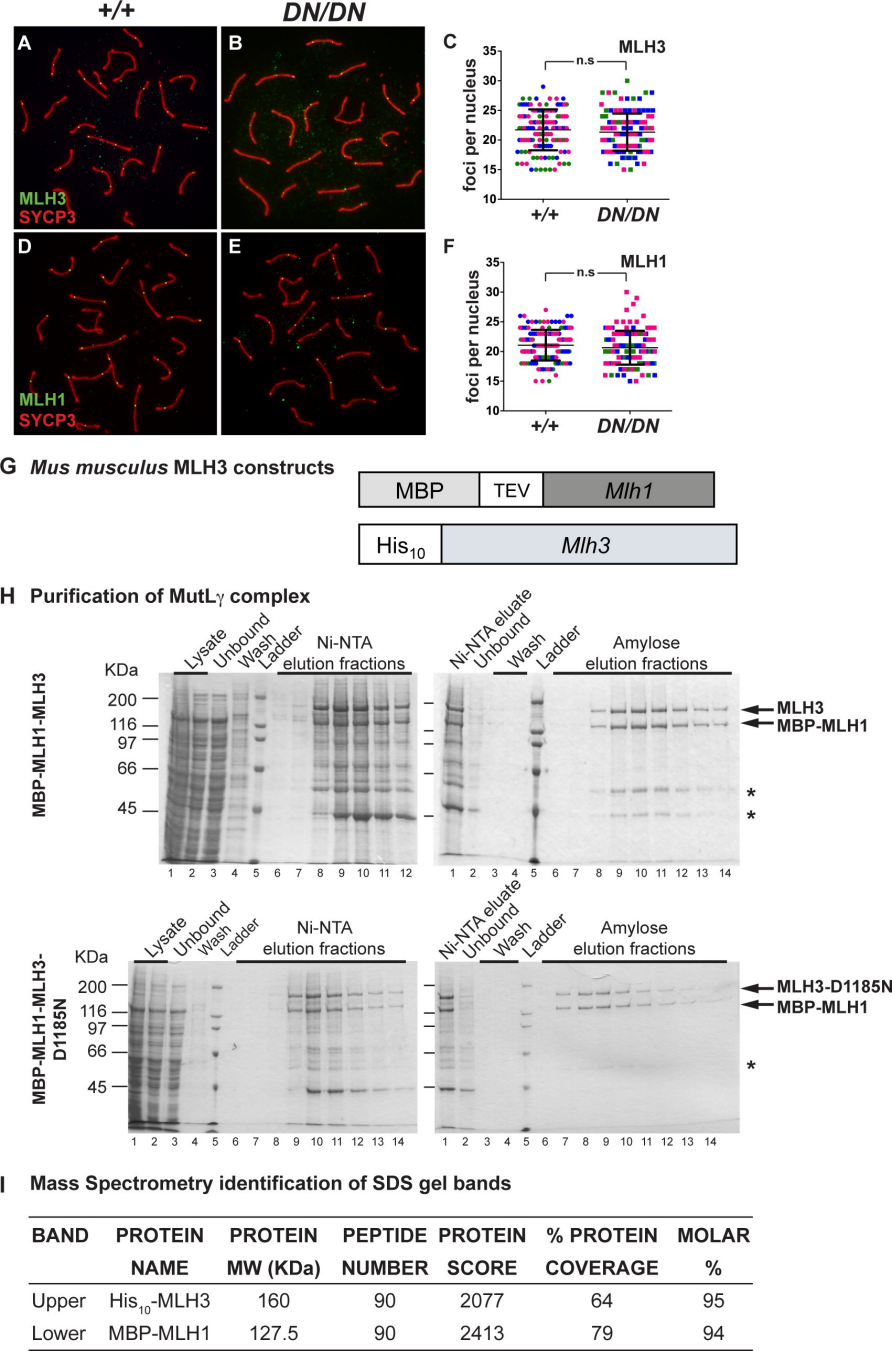
*Mlh3*^*DN/DN*^ pachytene spermatocytes exhibit normal localization of MutLγ while the recombinant MLH3-D1185N protein can form complexes with MLH1. (A-C) MLH3 (green) localizes to SYCP3 (red) in WT and *Mlh3*^*DN/DN*^ pachytene spermatocytes with no statistical difference in the number of MLH3 foci (WT = 21.7 ± 3.4 MLH3 foci n = 131, *Mlh3*^*DN/DN*^ = 21.3 ± 3.1 MLH3 foci n = 122; p = 0.36 by unpaired t-test). (D-F) MLH1 (green) localizes to SYCP3 (red) in WT and *Mlh3*^*DN/DN*^ pachytene spermatocytes also with no statistical difference in the number of MLH1 foci (WT = 21.1 ± 2.6 MLH1 foci n = 144, *Mlh3*^*DN/DN*^ = 20.2 ± 2.8 MLH1 foci n = 142; p = 0.2 by unpaired t-test). Different colors in (C) and (F) indicate 3 sets of matched littermates used, each color referring to the counts on a single animal (and color set from the two genotypes being processed simultaneously); n.s = not significant; error bars show standard deviation (s.d.). Note that for MLH1 and MLH3 counts, foci on the pseudoautosomal region (PAR) of the XY bivalent were excluded from quantitation. (G-I) MLH1-mlh3-D1185N forms a stable heterodimer. (G) Schematic of mouse *Mlh1* and *Mlh3* constructs (see Methods for details). (H) Representative purification of MBP-MLH1-MLH3 (top) and MBP-MLH1-mlh3-D1185N (bottom) using Ni-NTA and amylose resin chromatography as described in the Methods. Fractions were analyzed using SDS-PAGE, stained by Coomassie brilliant blue. MLH1-MLH3 and MLH1-MLH3-D1185N were eluted from amylose in the same fractions. The mass of molecular weight standards is indicated on the left and the expected positions of MBP-MLH1 (127.5 KDa) and His_10_-MLH3 (165 KDa) is indicated in the center. *Likely to be degradation products of MLH1-MLH3. (I) Mass spectrometry analysis of the two major bands in SDS-PAGE detected after amylose chromatography.

MutLγ represents the ultimate marker of DSB repair events that have adopted a class I CO fate, and has been used as a CO proxy marker in many organisms [29,62–64]. We anticipated that the D1185N endonuclease mutation in MLH3 would not affect localization of this complex. In WT spermatocytes, MLH3 localizes on the chromosomes during early pachynema, remaining associated with SYCP3 signal through to diplonema (Fig 4A). In pachytene spermatocyte preparations from *Mlh3*^*DN/DN*^ mice, MLH3 signal remains associated with the autosomal chromosome cores from early pachynema at a focus frequency that is statistically indistinguishable from that of WT cells (Fig 4A–4C, p = 0.36 by unpaired t-test). MLH3 association with the PAR of the synapsed X and Y chromosomes was similarly unaffected in *Mlh3*^*DN/DN*^ pachytene spermatocytes. In addition, the timing of MLH3 appearance, in early pachynema and prior to that of MLH1, was normal in *Mlh3*^*DN/DN*^ pachytene spermatocytes.

Localization of MLH1 was similarly explored in spermatocytes from *Mlh3*^*DN/DN*^ mice. As with MLH3, there was no difference in the timing of MLH1 accumulation on chromosome cores between WT and *Mlh3*^*DN/DN*^ mice (Fig 4D–4F). Moreover, when autosomal MLH1 foci were quantified, no statistical difference was observed in MLH1 focus frequency between WT and *Mlh3*^*DN/DN*^ pachytene cells (Fig 4D–4F, p = 0.2 by unpaired t-test). These data suggest that disruption of the endonuclease domain of MLH3 does not alter recruitment of MutLγ to chromosomes in pachynema.

### Normal localization of Class I Pro-CO factors in pachytene spermatocytes from *Mlh3*^*DN/DN*^ adult male mice

**Fig 5:**
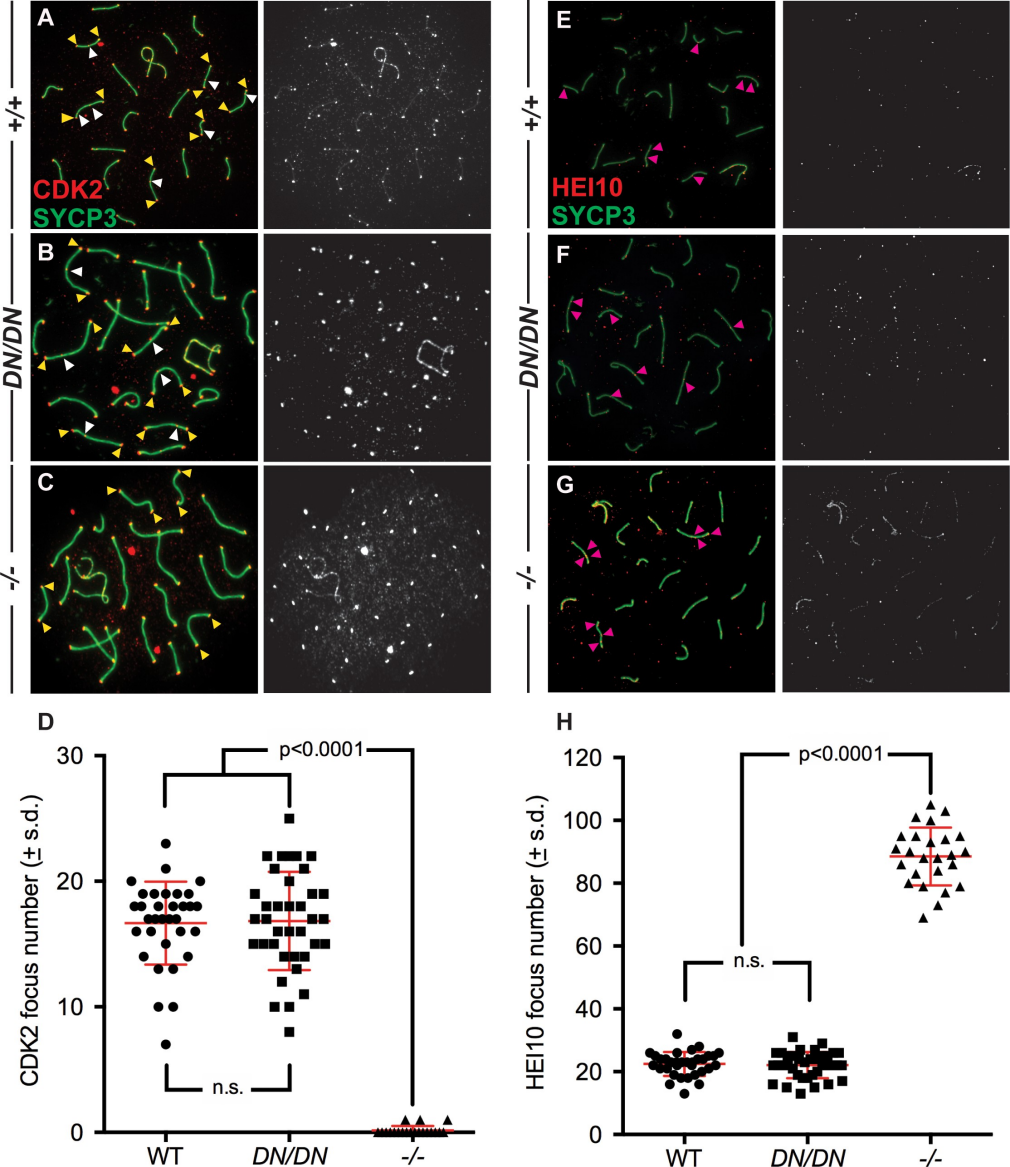
Normal localization of CDK2 and HEI10 to nascent sites of Class I crossovers in *Mlh3*^*DN/DN*^ spermatocytes. (A-D) CDK2 (red) localizes to the synaptonemal complex SYCP3 (green) in WT (A) and *Mlh3*^*DN/DN*^ (B) pachytene spermatocytes, but not in *Mlh3*^*-/-*^ cells (C), except at the telomeres. Left-hand panels show merged red and green channels, in which white arrows show examples of crossover associated CDK2, while yellow arrows show examples of telomere associated CDK2. Right-hand panels show only the CDK2 signal in white. Panel (D) shows quantitation of foci for each genotype at pachynema. Counts for WT and *Mlh3*^*DN/DN*^ are not statistically different from each other (by unpaired t-test with Welch’s correction). Values given are number of foci per nucleus ± s.d. (E-H) HEI10 (red) co-localizes with the synaptonemal complex SYCP3 (green) in pachytene spermatocytes from WT (E), *Mlh3*^*DN/DN*^ (F), and *Mlh3*^*-/-*^ (G) males. Left-hand panels show merged red and green channels, in which pink arrows show examples of crossover associated HEI10. Right-hand panels show only the HEI10 signal in white. For all chromosome imaging and foci counts, at least 3 mice of each genotype were observed for each staining set. Panel (H) shows quantitation of foci for each genotype at pachynema. Counts for WT and *Mlh3*^*DN/DN*^ are not statistically different from each other (by unpaired t-test with Welch’s correction). Values given are number of foci per nucleus ± s.d.

To observe class I CO events in pachynema, we employed two well characterized markers of these sites: the putative ubiquitin E3 ligase, Human Enhancer of Invasion-10 (HEI10), and cyclin-dependent kinase-2 (CDK2) [50,65,66]. In WT prophase I cells, CDK2 localizes to the telomeres (Fig 5A, yellow arrows) as well as on the chromosome cores (Fig 5A, white arrows) during mid to late pachynema and remains associated with SYCP3 signal through to diplonema [66]. The localization of CDK2 along chromosome cores parallels the localization of MLH1 and MLH3, both temporally and quantitatively (Fig 5A and 5D), and is associated with nascent class I CO events. In pachytene spermatocyte preparations from *Mlh3*^*DN/DN*^ mice, CDK2 signal remains associated with both the telomeres and chromosome cores at a frequency and intensity that is reminiscent of that seen in WT spermatocyte spreads (Fig 5B and 5D). This is in contrast to the situation in spermatocyte preparations from *Mlh3*^*-/-*^ males, in which CDK2 association with the telomere persists, but is lost from nascent CO sites (Fig 5C and 5D).

HEI10 was recently shown to co-localize with MutLγ at sites of class I CO, and its localization is dependent on Cyclin N-terminal Domain-containing-1 (CNTD1)[50,61]. HEI10 is thought to play a key role in CO designation/maturation [50]. As previously reported for WT cells at pachynema, HEI10 localizes with similar frequency to that of CDK2 and MutLγ (Fig 5E [pink arrows], 5H). Similar localization patterns and frequency were observed for *Mlh3*^*DN/DN*^ mice, with a frequency of one to two foci per chromosome (Fig 5F [pink arrows] and H), indicating normal recruitment of HEI10 on pachytene chromosome cores in *Mlh3*^*DN/DN*^ males. This is in contrast to the pattern of HEI10 staining in spermatocytes from *Mlh3*^*-/-*^ mice, where there is an increased accumulation of HEI10 foci (Fig 5G [pink arrows], 5H), as previously reported [50]. For both CDK2 and HEI10, the altered frequencies of foci observed in spermatocytes from *Mlh3*^*-/-*^ mice were significantly different from that of WT or *Mlh3*^*DN/DN*^ males (Welch’s T-test, p<0.0001). CDK2 and HEI10 focus counts for WT or *Mlh3*^*DN/DN*^ males were not statistically different from each other. Taken together, these observations demonstrate that loading of HEI10 and CDK2 on class I CO designated sites is affected differently by mutation of *Mlh3*: complete loss of MLH3 results in failure to load CDK2 and hyper-accumulation of HEI10, while altered endonuclease activity of MLH3 results in normal loading of both CDK2 and HEI10. Thus, the physical accumulation of MutLγ is required for normal loading of associated pro-crossover maturation factors.

### MLH1-MLH3-D1185N forms a stable heterodimer and displays similar chromatographic properties to MLH1-MLH3

Mouse *Mlh1* and *Mlh3* were amplified from cDNA and cloned into pFastBac1 vectors as described in the Methods. The MLH1-MLH3 and MLH1-MLH3-D1185N complexes were expressed from Sf9 cells infected with baculoviruses containing *MBP-Mlh1* and *His*_*10*_*-Mlh3* or *His*_*10*_*-Mlh3-D1185N* constructs (Fig 4G). Extracts from these cells were applied to a Ni-NTA column. Fractions containing induced proteins were pooled and then applied to an amylose column. Two major bands of molecular weights predicted for an MBP-MLH1-His_10_-MLH3 complex were detected on SDS-PAGE after amylose chromatography (Fig 4H). These bands were further analyzed by mass spectrometry, and the results from this analysis confirmed their identity (Fig 4I). Importantly, MLH1-MLH3 and MLH1-MLH3-D1185N eluted with an apparent 1:1 stoichiometry in both chromatography steps, indicating that the heterodimers were stable, and the protein yields of the two complexes after amylose chromatography were similar (Fig 4H).

### *Mlh3*^*DN/DN*^ diakinesis staged spermatocytes exhibit significantly fewer chiasmata than WT spermatocytes, but elevated chiasmata compared to *Mlh3*^*-/-*^ males

**Fig 6:**
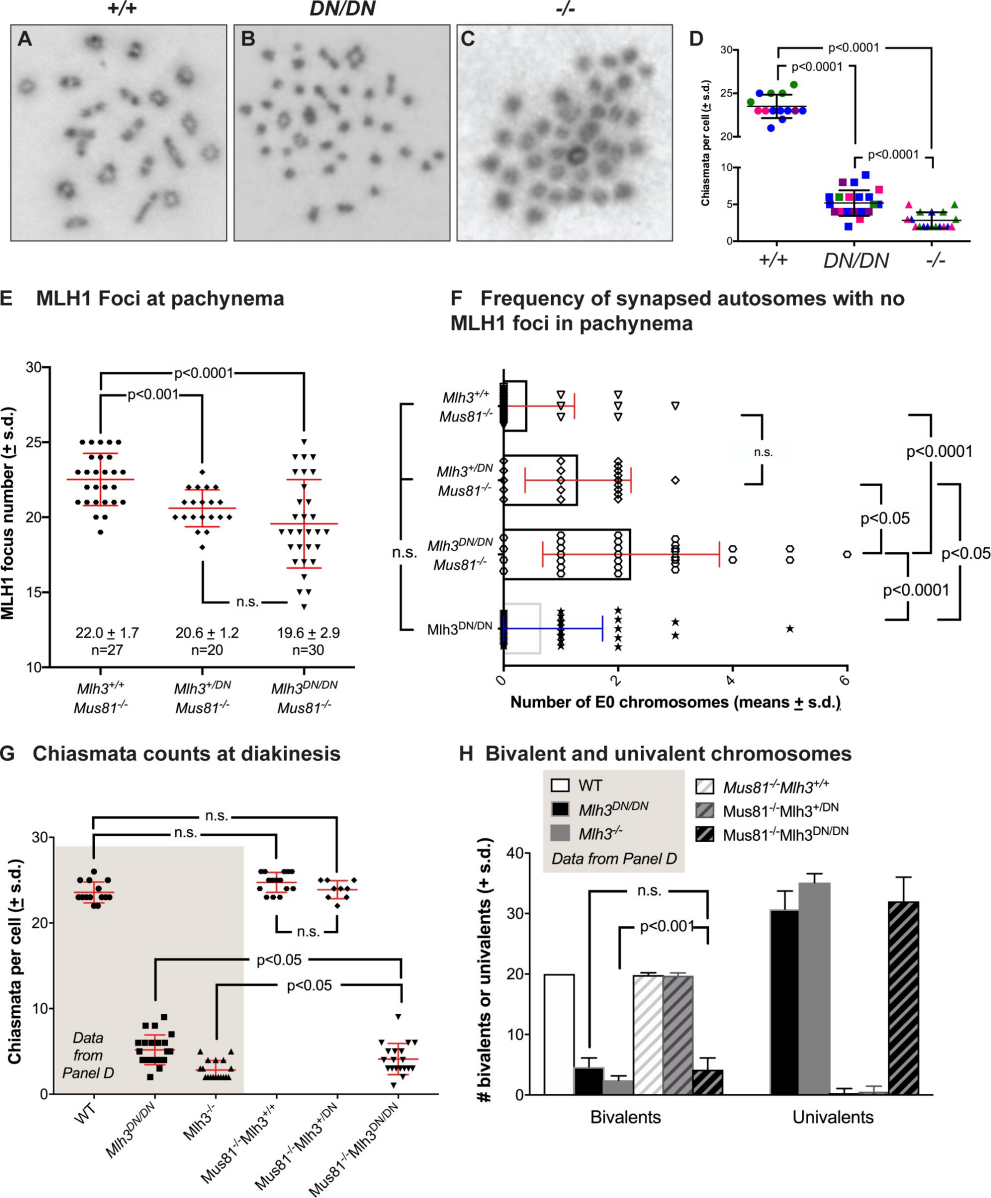
***Mlh3***^***DN/DN***^ **diakinesis staged spermatocytes show a reduced number of chiasmata (A-D)**, **while comparison of MLH1 focus frequency (E) and distribution (F) during pachynema, and chiasmata counts (G) and bivalent/univalent frequencies (H) in *Mus81***^***-/-***^***Mlh3***^***+/+***^**, *Mus81***^***-/-***^***Mlh3***^***+/DN***^**, and *Mus81***^***-/-***^***Mlh3***^***DN/DN***^ **males indicates partial involvement of class II CO pathway.** (A-C) Diakinesis staged spermatocyte preparations from WT, *Mlh3*^*DN/DN*^, and *Mlh3*^*-/-*^ males stained with Giemsa showing chiasmata formation between homologous chromosomes. Note that diakinesis spread chromosomes are not provided with scale bars because the degree of “spread” varies from cell to cell, thus it is hard to compare true magnifications between each image. Cells were viewed using a 100x objective. (D) *Mlh3*^*DN/DN*^ cells exhibit significantly fewer chiasmata when compared to WT (WT = 23.5 ± 1.3 chiasmata per nucleus, *Mlh3*^*DN/DN*^ = 5.2 ± 1.7; p values indicated on graph, by unpaired t-test with Welch’s correction, and using Bonferroni’s adjustment for multiple comparisons). *Mlh3*^*-/-*^ cells have significantly fewer chiasmata when compared to WT and *Mlh3*^*DN/DN*^ *(Mlh3*^*-/-*^ = 2.8 ± 1.1 chiasmata per nucleus; p < 0.0001 by unpaired t-test). All values are means ± s.d.. (E-H). We compared MutLγ frequency and distribution during pachynema between mice lacking *Mus81* with or without co-incident loss of a endonuclease-intact *Mlh3* allele (E, F). *Mus81*^*-/-*^*Mlh3*^*+/+*^ males (E; filled octagons) show elevated MLH1 focus frequency compared to mice bearing one or two copies of the *Mlh3*^*DN*^ allele (filled diamonds and down triangles, respectively). The reduced MLH1 focus count in *Mus81*^*-/-*^*Mlh3*^*+/DN*^, and *Mus81*^*-/-*^*Mlh3*^*DN/DN*^ males is statistically significant (p values indicated on graph, by unpaired t-test with Welch’s correction, and using Bonferroni’s adjustment for multiple comparisons). (F) The distribution of MLH1 foci is also disrupted in *Mus81*^*-/-*^*Mlh3*^*+/DN*^, and *Mus81*^*-/-*^*Mlh3*^*DN/DN*^ males, with increased numbers of synapsed autosomes showing no MLH1 foci, indicative of non-exchange (E0) chromosome pairs, compared to that seen in *Mus81*^*-/-*^*Mlh3*^*+/+*^ males (p values indicated on graph, by unpaired t-test with Welch’s correction, and using Bonferroni’s adjustment for multiple comparison). (G, H) the outcome of class I and class II CO events was assessed by quantifying chiasmata (G) and intact bivalent pairs (H) in diakinesis preparations from mice of all three double mutant genotype combinations, compared to single mutants presented in panel C, above. (G) Chiasmata counts were similar to WT for *Mus81*^*-/-*^*Mlh3*^*+/+*^ and *Mus81*^*-/-*^*Mlh3*^*+/DN*^ males (filled octagons and diamonds, respectively), but were statistically significantly lower in *Mus81*^*-/-*^*Mlh3*^*DN/DN*^ animals (filled down triangles). The frequency of chiasmata in *Mus81*^*-/-*^*Mlh3*^*DN/DN*^ animals was significantly lower than that observed in *Mlh3*^*DN/DN*^ animals, but significantly higher than that observed in *Mlh3*^*-/-*^ animals (p<0.05, unpaired t-test with Welch’s correction). However, the number of bivalent structures observed at diakinesis in *Mus81*^*-/-*^*Mlh3*^*DN/DN*^ animals (panel H) was unchanged from that observed in *Mlh3*^*DN/DN*^ animals. In all cases, two animals were assessed for each genotype. Note that for MLH1 and MLH3 counts, foci on the pseudoautosomal region (PAR) of the XY bivalent were excluded from quantitation.

Chiasmata are the physical manifestations of crossing over and, as such, can inform the process of DSB repair via all pathways. Diakinesis-staged spermatocytes from WT and *Mlh3*^*DN/DN*^ males were used to quantify chiasmata. WT cells exhibited a chiasmata frequency of 23.5 ±1.3 per nucleus (Fig 6A and 6D) whereas *Mlh3*^*DN/DN*^ spermatocytes exhibited a dramatically reduced chiasmata count of 5.2 ± 1.7 chiasmata per nucleus (Fig 6B and 6D; p < 0.0001 by unpaired t-test). Chiasmata counts for *Mlh3*^*-/-*^ males were even more dramatically reduced at 2.8 ± 1.1 chiasmata per nucleus, a value that is significantly lower than both WT and *Mlh3*^*DN/DN*^ spermatocytes (Fig 6C and 6D; p <0.0001 by unpaired t-test). Thus, complete loss of MLH3 protein leads to the loss of approximately 88% of chiasmata, while loss of endonuclease activity, but retention of MutLγ heterodimer results in only a 78% loss. Thus, the number of residual chiasmata observed in *Mlh3*^*DN/DN*^ spermatocytes is higher than the expected number of chiasmata achieved through the MUS81-EME1-driven class II CO pathway (~2–3, assessed both cytologically and genetically; [8,28]).

### Loss of *Mus81* on the *Mlh3*^*DN/DN*^ background results in altered MutLγ distribution and reduces residual chiasmata compared to *Mlh3*^*DN/DN*^ single null males

The increased residual chiasmata observed in *Mlh3*^*DN/DN*^ males compared to *Mlh3*^*-/-*^ animals prompted us to ask whether some or all of these crossovers were dependent on the activity of the MUS81-EME1 heterodimer. Previous studies in our lab showed that Mus81^-/-^ animals show increased accumulation of MutLγ, resulting in normal chiasmata counts, suggesting that class I CO events are up-regulated in the absence of the class II machinery [6]. Co-incident mutation of one or both *Mlh3* alleles to the *Mlh3*^*DN*^ variant on the *Mus81*^*-/-*^ mutant background yielded MLH1 focus counts that were significantly reduced compared to those observed in *Mlh3*^*+/+*^*Mus81*^*-/-*^ mice (Fig 6E, p<0.005 by unpaired t-test with Welch’s correction), and instead resembled MLH1 focus counts observed in spermatocytes from *Mlh3*^*+/+*^ mice (Fig 4F). Thus, the upregulation of MLH1 foci at pachynema requires both a defective MUS81-EME1 dimer and the presence of only functional MutLγ heterodimer.

Interestingly, pachytene spermatocytes from *Mlh3*^*+/DN*^ *Mus81*^*-/-*^ and *Mlh3*^*DN/DN*^ *Mus81*^*-/-*^ males show an abnormal distribution of MLH1 foci across all autosomal pairs (Fig 6F). In *Mlh3*^*+/+*^*Mus81*^*-/-*^ males, 17% (5/29) cells showed synapsed chromosomes without any MLH1 foci in pachynema (so-called “no exchange” or “E0” chromosomes), while in *Mlh3*^*+/DN*^ *Mus81*^*-/-*^ males, this proportion increased to 67% (8/12), with up to 3 E0 chromosomes per cell (Fig 6F; examples shown in S5 Fig). In *Mlh3*^*DN/DN*^*Mus81*^*-/-*^ males, 94% of cells had E0 chromosomes (29/31), with as many as 6 E0 bivalents being observed (Figs 6F and S5). The higher proportion of MLH1-devoid autosomes in *Mlh3*^*+/DN*^ and *Mlh3*^*DN/DN*^ males on the *Mus81* null background was statistically significant in all pairwise comparisons (Fig 6F, unpaired t-test with Welch’s correction with Bonferroni adjustment), indicating that the placement of an obligate crossover is perturbed in mice having one or two copies of the *Mlh3*^*DN*^ allele on a *Mus81* null background, and suggesting that mechanisms that ensure correct CO placement require a function MutLγ complex. In yeast, and also probably in mice, CO interference requires a functional MutSγ complex [67]. However, MutSγ alone is not sufficient to ensure appropriate CO placement since *Mus81*^*-/-*^ males exhibit disrupted interference despite appropriate MutSγ loading [6].

Assessment of chiasmata counts in single (Fig 6D) and double mutants (Fig 6G) revealed the expected normal chiasmata frequency in spermatocytes from *Mus81*^*-/-*^*Mlh3*^*+/+*^ and *Mus81*^*-/-*^*Mlh3*^*+/DN*^ males, and the loss of most chiasmata in spermatocytes from *Mus81*^*-/-*^*Mlh3*^*DN/DN*^ males. However, whereas the loss of chiasmata structures in *Mlh3*^*-/-*^ and *Mlh3*^*DN/DN*^ single mutants was observed to be 88% and 78%, respectively (Fig 6D), the loss of chiasmata in *Mus81*^*-/-*^*Mlh3*^*DN/DN*^ males was 83%. The frequency of chiasmata in cells from these double mutants was statistically different from both *Mlh3* single homozygous mutant animals (p<0.05, unpaired t-test with Welch’s correction). Thus, loss of the class II pathway in addition to the mutation of the endonuclease domain of *Mlh3* only partially reduces residual chiasmata counts to the levels observed in *Mlh3*^*-/-*^ animals. Interestingly, despite reduced chiasmata, the overall number of bivalent structures observed in diakinesis preparations from *Mus81*^*-/-*^*Mlh3*^*DN/DN*^ males is the same as that seen in *Mlh3*^*DN/DN*^ males, and is significantly elevated above that seen in *Mlh3*^*-/-*^ single mutant males (Fig 6H). Taken together, these observations suggest two important features regarding class I/II interactions: (1) that a fully functional class I and class II machinery is required for appropriate distribution of MutLγ foci across the genome; and 2) that MUS81-EME1 activity cannot fully account for the residual chiasmata count observed in *Mlh3*^*DN/DN*^ males.

## Discussion

Studies in *S*. *cerevisiae* and *M*. *musculus* have implicated MutLγ as the major resolvase of dHJs in the class I CO pathway [29,32–35,41,43,44,68]. The current study examines the importance of an intact endonuclease domain for the proper functioning of MLH3 during prophase I of mammalian meiosis, and is the first exploration of a point mutation for MutLγ in the mouse. We generated a mouse with a mutation in the MLH3 endonuclease domain that affects its catalytic activity while allowing for heterodimer assembly. We found that, as in *Mlh3*^*-/-*^ mutant males, *Mlh3*^*DN/DN*^ males are infertile, exhibit significantly smaller testes than their WT litter mates, and have no epididymal spermatozoa. Beyond this, our data reveal important similarities and differences in the meiotic phenotypes to that observed with a nullizygous *Mlh3*^*-/-*^ allele, as articulated below.

Importantly, the phenotypic consequences of loss of a functional MutLγ complex occur despite normal accumulation of both MutSγ and MutLγ indicating that the physical presence of these complexes is not sufficient to ensure complete CO resolution. However, CO licensing (defined by MutSγ deposition) and designation (defined by MutSγ and MutLγ deposition) are both normal in *Mlh3*^*DN/DN*^ males, indicating that a functional MutLγ complex is not required for these CO-defining processes. Similarly, although normal accumulation of pro-crossover factors, CDK2 and HEI10, is observed in *Mlh3*^*DN/DN*^ males (unlike the situation in *Mlh3*^*-/-*^ males), this is not sufficient to drive CO resolution along the class I CO pathway.

We demonstrate that an intact endonuclease domain within MLH3 is not required for DSB or synaptonemal complex formation in early prophase I, similar to that seen in *Mlh3*^*-/-*^ males [29]. However, there are distinct differences in RAD51 accumulation and persistence in spermatocytes from *Mlh3*^*-/-*^ and *Mlh3*^*DN/DN*^ males, suggesting that the effect of MutLγ loss on DSB repair processing is quite different from the presence of a defective MutLγ complex. Most importantly, while RAD51 is recruited in elevated numbers to chromosome cores of *Mlh3*^*DN/DN*^ males, it fails to be cleared effectively in pachynema, perhaps because the defective MutLγ complex blocks subsequent processing of DSB repair intermediates. Intriguingly, the significantly altered RAD51 accumulation in leptonema in both *Mlh3* mutants indicates a role for MutLγ prior to pachynema, far earlier than has been defined thus far. Indeed, analogous early pairing roles for MutLγ have been proposed for *M*. *sordaria* and *S*. *cerevisiae* [42,53]. In yeast, Al-Sweel *et al*. constructed whole genome recombination maps for wildtype, endonuclease defective, and null *mlh3* yeast mutants. Both the endonuclease defective and null yeast mutants for *mlh3* showed increases in the number of NCO events, consistent with recombination intermediates being resolved through alternative recombination pathways [34]. Thus, in the case of yeast, loss of Mlh3 protein, or the production of an endonuclease defective protein, increases the frequency of other recombination outcomes, most notably including earlier NCO events.

The absence of either component of MutLγ results in the loss of 90–95% of chiasmata, consistent with the established dogma that class I COs account for the majority, but not all, chiasmata in mammalian meiosis [6,8,29,39,68]. By contrast, loss of MUS81, the major class II CO regulator, results in normal chiasmata levels as a result of up-regulation of class I events, as evidenced by a ~10% increase in MutLγ localization during pachynema [6], suggesting that loss of the class II pathway leads to a compensatory increase in class I events. Furthermore, our previous analysis of *Mlh3*^*-/-*^*Mus81*^*-/-*^ double mutant animals revealed a very small (<1 on average), but consistent, number of residual chiasmata, indicating the existence of other resolvase complexes [6], as has been demonstrated for yeast and plants [10,11]. Taken together, these observations have two important implications for crossing over in the mouse: first, additional class I CO events can be achieved through recruitment of additional MutSγ-designated precursor sites in the absence of the class II pathway (and possibly under other circumstances too), and second, a few crossovers can be achieved without implementing either MutLγ or MUS81-EME1.

In the current study, we show that spermatocytes from *Mlh3*^*DN/DN*^ males show normal accumulation of both MLH1 and MLH3 at pachynema, but this results in an increase in the residual chiasmata count at diakinesis relative to that seen in *Mlh3*^*-/-*^ mice: approximately 78% of COs are lost in *Mlh3*^*DN/DN*^ spermatocytes, leading to 22% residual chiasmata. This suggests several possibilities: either that the endonucleolytic function of MLH3 does not account for the resolution of all class I COs under wildtype situations, and/or that other resolvases can be recruited under certain circumstances once MutLγ loads, irrespective of whether this complex is endonucleolytically competent. Alternatively, the point mutation in the endonuclease domain does not completely eliminate endonucleolytic activity in the mouse, resulting in partial class I resolvase activity. We find this latter possibility unlikely due to the severity of the defect in endonuclease activity in the *S*. *cerevisiae* Mlh1-mlh3-D523N complex [32,33], but we were unable to test this in the current analysis.

Another explanation for the difference in chiasmata counts between *Mlh3*^*DN/DN*^ and *Mlh3*^*-/-*^ mice is that, in the former, the existence of a defective MutLγ prevents most class I-type COs, but facilitates the resolution of recombination intermediates through alternative pathways. Thus, despite the presence of the MLH3^DN^ protein, some class I CO events can be processed by other CO machinery under conditions of normal accumulation of pro-crossover factors, MutSγ, HEI10 and CDK2. While we cannot assess the recruitment of the class II machinery to sites of DSB repair in prophase I in the mouse (due to the lack of available reagents), there is evidence to support the idea that MUS81-EME1 might participate in this CO resolution crosstalk. Specifically, double mutants lacking *Mus81* and bearing a homozygous *Mlh3*^*DN*^ allele show reduced chiasmata relative to *Mlh3*^*DN/DN*^ males, indicating that MUS81 may account for at least some of the increase in chiasmata above that of *Mlh3*^*-/-*^ males. Arguing against this idea is the observation that the loss of *Mus81* on the *Mlh3*^*-/-*^ background results in a similar drop in residual chiasmata from *Mlh3*^*-/-*^ single mutant males alone to that observed in *Mlh3*^*DN/DN*^*Mus81*^*-/-*^ double mutant animals compared to *Mlh3*^*DN/DN*^ single mutant animals.

Previous analysis of *Mus81*^*-/-*^ single mutant males revealed a compensatory increase in MutLγ in pachynema that resulted in normal crossover numbers as a result of upregulation of class I CO events [6]. The persistence of RAD51 at several foci in late pachynema in these *Mus81*^*-/-*^ males suggested that the class II-destined DSBs were not repaired, but that additional COs were derived from increased CO designation from the larger pool of MutSγ-loaded DSB repair intermediates, suggesting some crosstalk between CO pathways to achieve CO homeostasis. Further evidence for crosstalk between the two major crossover pathways is provided in the current study. However, this elevated MLH1 focus frequency is not observed in *Mlh3*^*DN/DN*^*Mus81*^*-/-*^ and *Mlh3*^*+/DN*^*Mus81*^*-/-*^ males, suggesting that a functional MutLγ is required for this crosstalk. Intriguingly, loss of *Mus81* on the *Mlh3*^*DN/DN*^ and *Mlh3*^*+/DN*^ backgrounds results in altered distribution of MLH1 across the genome, resulting in elevated numbers of chromosomes lacking an MLH1 focus entirely. This increase in “E0” chromosomes does not occur in either *Mus81*^*-/-*^ or *Mlh3*^*DN/DN*^ single null males, indicating that CO distribution is dependent on the functionality (or partial functionality) of both pathways. While we cannot fully explain the reason for altered MLH1 distribution in both *Mlh3*^*DN/DN*^*Mus81*^*-/-*^ and *Mlh3*^*+/DN*^*Mus81*^*-/-*^ males, these observations point to complex interplay between crossover pathways in achieving normal distribution of crossover events in mammalian meiosis. Indeed, our recent studies involving a mouse model harboring a point mutation within *Msh5* indicates that altered MutSγ function affects both crossover pathways [69].

Our previous studies, along with the current one, indicate a role for BLM helicase in modulating the pathway choice in DSB repair during mouse meiosis. In *Mlh3*^*-/-*^ [56] and *Mlh3*^*DN/DN*^ (current work) males, prophase I spermatocytes show increased and persistent accumulation of BLM helicase through until late pachynema. A similar increase in BLM localization was also noted in *Mus81*^*-/-*^ spermatocytes [6]. In *S*. *cerevisiae*, loss of class I CO pathway components (for example, in *msh4/5* or *mlh1/3* mutants) is suppressed by mutation of the BLM ortholog, *Sgs1*, highlighting the role of Sgs1 as an anti-crossover factor. However, the additional CO events that arise in these double mutant yeast strains are presumed to be processed via Mus81-dependent resolution, and thus via class II CO events [13,36,70]. In *msh4/5 sgs1* double mutant strains, the restoration of COs occurs without any concomitant decrease in NCO events, suggesting either that other CO pathways account for the non-class I COs, or that these DSBs are repaired via inter-sister repair processes. In this sense, Sgs1 has been proposed to be master orchestrator of recombination pathway choice [36], while the Sgs1-Top3-Rmi1 complex as a whole can regulate CO formation both positively and negatively in yeast [23,24]. The situation we observe in *Mlh3*^*-/-*^ and *Mlh3*^*DN/DN*^ males with respect to BLM persistence may be similar to that seen in yeast for Sgs1, in that up-regulation of BLM foci is observed in both *Mlh3* mutant lines from zygonema onwards, but is significantly higher in *Mlh3*^*DN/DN*^ males at pachynema. Thus, residual chiasmata counts in *Mlh3*^*-/-*^ and *Mlh3*^*DN/DN*^ males are proportional to pachytene BLM focus counts (lower in full nulls, higher in *Mlh3*^*DN/DN*^ males). Thus, we can postulate that the loss of MLH3 protein entirely in *Mlh3*^*-/-*^ males results in a compensatory, but ineffective, increase in BLM that cannot overcome the failure to process class I COs sufficiently (a situation that is different to yeast). In the presence of intact, but catalytically inert MutLγ, on the other hand, the availability of additional BLM foci can then direct DSB repair in favor of other CO pathways in a similar fashion to the situation in yeast, where the engagement of Sgs1 promotes alternative repair mechanisms, primarily through the recruitment of structure specific nucleases, and the resolution of some dHJs through a class II (or other) CO pathway [13,36,70–72].

We provide evidence that COs are achieved in *Mlh3*^*DN/DN*^ spermatocytes in a manner that may be dependent on the MUS81-EME1 endonuclease, or on other resolvase complexes that have yet to be determined in mammalian meiosis. Indeed, our previous analysis of *Mlh3*^*-/-*^*Mus81*^*-/-*^ males indicated the existence of additional CO events that were independent of the class I and class II pathways [6]. Additional resolvases in yeast include SLX1-SLX4 and YEN1/GEN1[35,73,74]. The persistence of DSB repair intermediates into pachynema, along with the upregulated and persistent BLM might suggest that the defective MutLγ complex prevents accumulation of other such resolvase complexes. This might, in turn delay CO maturation until later in prophase I when, for example, GEN1 can be invoked to resolve the CO [75]. Thus, we propose that the timing of MutLγ activity, and its clearance from nascent COs is an important factor in the recruitment of alternative CO processing machineries, but in a manner that is not dependent on its endonuclease activity.

## Materials and methods

### Ethics statement

Work performed in this manuscript was approved by the Cornell Institutional Animal Care and Use Committee, under protocol 2004–0063.

### Generation of mice and genotyping

A PL253 targeting vector containing the *Mlh3-D1185N* point mutation in the potential endonuclease domain and a loxP-neo-loxP cassette in intron 5–6 of *Mlh3* was incorporated into an embryonic stem cell line. *Mlh3*^*DN*^ transgenic mice were crossed with a *Spo11-Cre* mouse line to remove the *neo* cassette [76], and then maintained on an inbred background through backcrossing on to the C57Bl/6J line (Jackson Laboratory, Bar Harbor, ME). Genotyping of WT, *Mlh3*^*+/DN*^, and *Mlh3*^*DN/DN*^ mice was performed using the following PCR primer pairs: forward (5’-AAGCCAAGTCTGCATGAGTA-3’) and reverse (5’-TAAATGTGCCACTGACTAAAT-3’) followed by a restriction enzyme digestion with *Sau96I* (New England Biolabs) at 37°C for 2–3 hours, which results in 439-bp and 263-bp fragments from the WT allele and a 702-bp fragment from the mutant allele. Fertility tests were performed by breeding *Mlh3*^*DN/DN*^ adult males with WT females. At least 3 males of each genotyped were evaluated. Presence of a copulation plug the following morning counted as a successful mating event. Pregnancy was confirmed by gentle palpation of the abdomen after gestation day 11 or on delivery date of litters. *Mus81*^*-/-*^ animals were generated from our breeding stock of such mice, as previously described [6]. Mice were housed and utilized under the guidance and approval of the Cornell University Institutional Animal Care and Use Committee.

### Histology

Testes from adult mice were fixed in Bouin’s solution overnight at room temperature and then washed 3 x 10 min with 70% ethanol at room temperature with agitation. Fixed and paraffin-embedded testes were section at 5 μm. H&E staining was performed on Bouin’s fixed testes using standard methods. At least 6 males of each genotyped were evaluated.

### Sperm counts

Caudal epididymides were removed from adult males and placed in pre-warmed 1X PBS containing 4% bovine serum albumin. Sperm were released into solution by squeezing epididymis with tweezers and incubated for 20 min at 32°C/5% CO_2_. After incubation, 20 μL of sperm suspension was re-suspended in 480 μL of 10% formalin. Sperm counts were performed with a hemocytometer. At least 10 males of each genotype were evaluated for sperm counts and testis weights.

### Prophase I chromosome analysis and immunofluorescence

Prophase I chromosome spreads from adult testes were prepared as previously described [28,61]. For all experiments, at least 6 males of each genotyped were evaluated. Chromosome slides were then washed in 0.4% Kodak Photo-Flo 200/1X PBS for 2 x 5 min, 0.4% Kodak Photo-Flo 200/dH_2_O for 2 x 5 min, then air-dried for approximately 10 min and stored in -80°C or used immediately for staining. Primary antibodies used were: anti-γH2AX (Millipore, NY, #05–636 1:10,000), anti-SYCP3 (Abcam, MA, #97672, 1:5000), anti-SYCP1 (Abcam, MA, #15087, 1:1000), anti-RAD51 (Calbiochem, #PC130, 1:500), anti-BLM (generous gift from Dr. Ramundo Freire; 1:100;), anti-CDK2 (Santa Cruz, TX, sc-163; 1:250), anti-MLH3 ([61]; 1:1000), anti-RNF212 (generous gift from Dr. Neil Hunter), anti-RPA (generous gift from Dr. Jeremy Wang; 1:500), anti-MSH4 (Abcam, MA, #58666; 1:500), anti-HEI10 (Anti-CCNB1IP1, Abcam, MA # 71977) and anti-MLH1 (BD Biosciences Pharmingen, CA, #550838, 1:100). Secondary antibodies used were: goat anti-mouse Alexa Fluor 488 (#62–6511), goat anti-mouse Alexa Fluor 555 (#A-10521), goat anti-rabbit Alexa Fluor 488 (#65–6111), goat anti-rabbit Alexa Fluor 555 (#A-10520; all Invitrogen, 1:2000).

### Spermatocyte diakinesis spread preparations to observe chiasmata

Diakinesis chromosome spreads were prepared as previously with slight modifications [61,77]. Slides were stained with 10% Giemsa for 10 mins, washed, air-dried and mounted with Permount.

### Image acquisition

All chromosome spread slides were visualized using the Zeiss Imager Z1 microscope (Carl Zeiss, Inc.). Images were captured with a high-resolution microscopy camera AxioCam MRM (Carl Zeiss, Inc.) and processed with ZEN Software (version 2.0.0.0; Carl Zeiss, Inc.).

### Quantitation of foci

Focus counts were performed manually by at least two people, and the results averaged before analysis. For RPA, we employed and ImageJ algorithm for automated counting, as described [69], and compared this automated calculation to manual counts for consistency. Manual and automated counts were not statistically significantly different to each other.

### Cloning mouse *Mlh1* and *Mlh3*

cDNA was synthesized from total testis RNA from wildtype C57B/6J adult males using the SuperScript III Reverse Transcriptase Kit from ThermoFisher. *Mlh1* and *Mlh3* open reading frames were PCR amplified from cDNA using Expand High Fidelity DNA polymerase using primer pairs AO3365 (5’GCTAGCAGCTGATGCATATGGCGTTTGTAGCAGGAG) and AO3366 (5’TACCGCATGCTATGCATTAACACCGCTCAAAGACTTTG) for *Mlh1*, and AO3367 (5’ACGTCGACGAGCTCATATGCATCACCATCACCATCACCATCACCATCACATCAGGTGTCTATCAGATGAC) and AO3368 (5’CGAAAGCGGCCGCGATCATGGAGGCTCACAAGG) for *His*_*10*_-*Mlh3*. Each fragment was cloned into the *Spe*1 site of pFastBac1 (ThermoFisher) using Gibson assembly PCR (NEB) to create pEAE393 (*Mlh1*) and pEAE397 (*His*_*10*_*-Mlh3*). Constructs were verified by DNA sequencing with NCBI reference sequences NM_026810.2 and NM_175337.2 for *Mlh1* and *Mlh3*, respectively. These constructs were then modified as follows:

1. The MBP–TEV sequence was inserted at the N-terminus of *Mlh1* in pEAE393 to create pEAE395.
2. The *mlh3-D1185N* mutation was introduced into pEAE397 by Quick Change (Agilent) to create pEAE413.

### Chromatography analysis of the mouse MLH1-MLH3-D1185N heterodimer from Baculovirus-infected Sf9 cells

*Sf9* cells were transfected with pEAE397 (*His*_*10*_*-Mlh3*), pEAE413 (*His*_*10*_*-Mlh3-D1185N*) and pEAE395 (*MBP-Mlh1*) using the Bac-to-Bac baculovirus infection system (Invitrogen). Fresh *Sf9* cells were co-infected with both viruses (containing *Mlh1* and *Mlh3* or *Mlh3-D1185N*). Cells were harvested 60 hours post infection, washed with phosphate buffered saline, and kept at -80°C until use.

Cell pellets from 250 ml of cells as thawed, resuspended in 60 ml hypotonic lysis buffer (20 mM HEPES-KOH pH 7.5, 5 mM NaCl, 1 mM MgCl_2_, 1 mM PMSF and EDTA free protease inhibitor mixture from Roche and Thermo Scientific) and incubated for 15 min on ice. The suspension was adjusted to 250 mM NaCl, 15 mM imidazole, 10% glycerol, 2 mM ß-mercaptoethanol (BME), and clarified by centrifugation at 17,000 g for 20 min at 4°C. The supernatant was mixed with 6 ml of 50% nickel-nitrolotriaceticacid-agarose (Ni-NTA) resin and allowed to bind for 2 hours or overnight followed by centrifugation to remove the unbound fraction. The resin was packed onto a column and washed with 7–10 column volumes of wash buffer (50 mM HEPES-KOH pH 7.5, 250 mM NaCl, 40 mM imidazole, 10% glycerol, 2 mM BME, 1 mM PMSF). Protein was eluted with 15 ml of 300 mM imidazole in 50 mM HEPES-KOH pH 7.5, 250 mM NaCl, 40 mM imidazole, 10% glycerol, 2 mM BME and 1 mM PMSF. Elution fractions containing MLH1-MLH3, determined by SDS-PAGE, were pooled and loaded onto 1 ml 100% amylose resin (NEB). The resin was washed with 10 column volumes of wash buffer (50 mM HEPES-KOH pH 7.5, 250 mM NaCl, 10% glycerol, 2 mM BME, 1 mM PMSF) and eluted with 6 ml wash buffer containing 10 mM maltose. Fractions containing MLH1-MLH3 were pooled and aliquots were flash frozen and stored in -80°C. The protein yield, following amylose chromatography, was similar for wild-type and mutant complexes (approximately 120–150 μg per 250 ml cells).

It is important to note that we were unable to detect a specific endonuclease activity for the mouse MBP-MLH1-MLH3 complex, suggesting that the MBP tag interferes with MLH1-MLH3 functions. We were unable to test this directly because, despite numerous attempts, we were unable to efficiently remove the MBP tag from MLH1 by treating MBP-MLH1-MLH3 with TEV protease.

### Mass-spectrometry of MLH1 and MLH3 bands from SDS-PAGE

SDS-PAGE bands following amylose chromatography predicted to contain MBP-MLH1 and His_10_-MLH3 were excised and analyzed by the Cornell University Proteomics facility using a Thermo LTQ Orbitrap Velos Mass Spectrometer.

### Statistical methods and analysis

The majority of comparisons involved with unpaired parametric t-test with Welch's correction or nonparametric Mann-Whitney U-test, depending on the data distribution. Where necessary, Bonferroni’s adjustment was used for multiple comparisons. All statistical analysis was performed with GraphPad Prism Version 7.00 for Mac, Graphpad Software, La Jolla California USA, www.graphpad.com. P-values less than 0.05 were considered statistically significant.

## Supporting information

S1 Fig Amino acid sequence of MLH3 endonuclease domain and its conservation across species. (A) Amino acid protein sequence of the *M*. *musculus*, *H*. *sapiens*, *S*. *cerevisiae*, *and A*. *thaliana* MLH3 endonuclease domain, DQHA(X)_2_E(X)_4_E, shows the conservation of this domain across these species. Asterisk refers to the conserved aspartic acid (D) that was targeted for a point mutation and converted to asparagine (N) to generate the *Mlh3-DN* mouse. (B) *Mus musculus* MLH3 is composed of a 1411 amino acid long sequence that results in an ~158 kDa sized protein (UniProt, 2015). MLH3 contains a globular N-terminal domain (NTD; light gray) and C-terminal domain (CTD; light blue) connected by a flexible linker arm (white). The NTD (light gray) contains ATP binding motifs (dark gray) that are conserved across species. The CTD consists of the MLH1 interacting domain (light blue) and the conserved endonuclease motif (dark blue). The aspartic acid (D) in the conserved endonuclease motif in mouse, DQHAAHERIRLE, was converted to an asparagine (N; red) at amino acid site 1185.
(TIF)

S2 Fig DSB formation and signaling as well as synapsis are normal in *Mlh3^DN/DN^* spermatocytes throughout prophase I. (A-O) *Mlh3*^*DN/DN*^ prophase I cells exhibit grossly normal DSB formation and signaling as observed by γH2AX staining (green) on synaptonemal complex protein SYCP3 (red) as compared to WT and *Mlh3*^*-/-*^ cells. Images show abundant γH2AX signal in leptonema, following by diminished signal in zygonema, with the absence of signal in pachynema and diplonema, except at the sex body, due to MSCI. (E, J, O) Over exposure of the γH2AX signal results in γH2AX foci or flares on the autosomes in WT, *Mlh3*^*DN/DN*^, as well as *Mlh3*^*-/-*^ cells (white arrows), suggesting that this signal may not be representative of true un-repaired DSBs. (P-W) *Mlh3*^*DN/DN*^ prophase I cells have normal synapsis as observed by the localization of synaptonemal complex protein SYCP1 (green) and SYCP3 (red) on the chromosomes when compared to WT. SYCP3 forms as short patches along the chromosomes in leptonema, extending into filaments in zygonema along with the appearance of SYCP1, full synapsis with the co-localization of SYCP1 and SYCP3 are observed in pachynema, followed by desynapsis in diplonema with the degradation of SYCP1.
(TIF)

S3 Fig RPA foci through prophase I in *Mlh3^+/+^* and *Mlh3^DN/DN^* spermatocytes. (A) Quantitation of RPA foci in spermatocytes from leptonema through diplonema shows initially elevated RPA numbers, with a progressive decline through prophase I for both WT and *Mlh3*^*DN/DN*^ spermatocytes. However, at leptonema and diplonema, the RPA focus counts are significantly elevated in *Mlh3*^*DN/DN*^ spermatocytes compared to WT littermate controls (p values given in graph: unpaired t-test with Welch’s correction). Values given are number of foci per nucleus ± s.d. (B-I) Example spread images of RPA staining at different prophase I stages, including leptonema (B,F), zygonema (C,G), pachynema (D,H), and diplonema (E,I) from *Mlh3*^*+/+*^ (B-E) and *Mlh3*^*DN/DN*^ (F-I) male mice. Chromosome spreads were stained with antibodies against SYCP3 (red) and RPA (green).
(TIF)

S4 Fig Normal localization of crossover designation factor MSH4 in *Mlh3^DN/DN^* and *Mlh3^-/-^* spermatocytes. (A-M) RNF212 (green) localization on chromosome cores stained with antibodies against SYCP3 (red) through early prophase I (leptonema, zygonema, early pachynema, and late pachynema). Representative images of spermatocytes from WT (A-D), *Mlh3*^*DN/DN*^ (E-H), and *Mlh3*^*-/-*^ (I-L) adult males. Panel M shows the quantitation of RNF212 foci in all three genotypes at early (EP) and late pachynema (LP). RNF212 accumulates on chromosome cores at zygonema in high numbers and these foci diminish gradually through pachynema with only one or two foci remaining in late pachynema. (N-Z) MSH4 (green) localization on chromosome cores stained with antibodies against SYCP3 (red) through early prophase I (leptonema, zygonema, early pachynema, and late pachynema). Representative images of spermatocytes from WT (M-P), *Mlh3*^*DN/DN*^ (Q-T), and *Mlh3*^*-/-*^ (U-X) adult males. Panel Z shows the quantitation of MSH4 foci in all three genotypes at early (EP) and late pachynema (LP). A similar pattern of MSH4 foci accumulation and loss is observed for MSH4 as for RNF212: accumulation of high numbers of foci in zygonema, diminishing to one or two foci per chromosome in late pachynema. In all cases, statistical analysis was performed using unpaired t-test with Welch’s correction (p values provided in graphs), with Bonferroni’s adjustment for multiple comparisons where necessary. For all chromosome imaging and foci counts, at least three mice of each genotype were observed for each staining set.
(TIF)

S5 Fig Examples of pachytene chromosome spreads from *Mlh3^DN/DN^Mus81^-/-^* males showing chromosomes with no MLH1 foci. E0 chromosomes are highlighted with a white box. The XY bivalent is indicated (but was not included in the counts because the MLH1 focus is not always visible on the PAR).
(TIF)
