# Supplementary figures and images for: A mutation in the endonuclease domain of mouse MLH3 reveals novel roles for MutLγ during crossover formation in meiotic prophase I
Source: PLoS Genet. 2019 Jun 6;15(6):e1008177. doi: 10.1371/journal.pgen.1008177 (PMC6588253; doi:10.1371/journal.pgen.1008177)

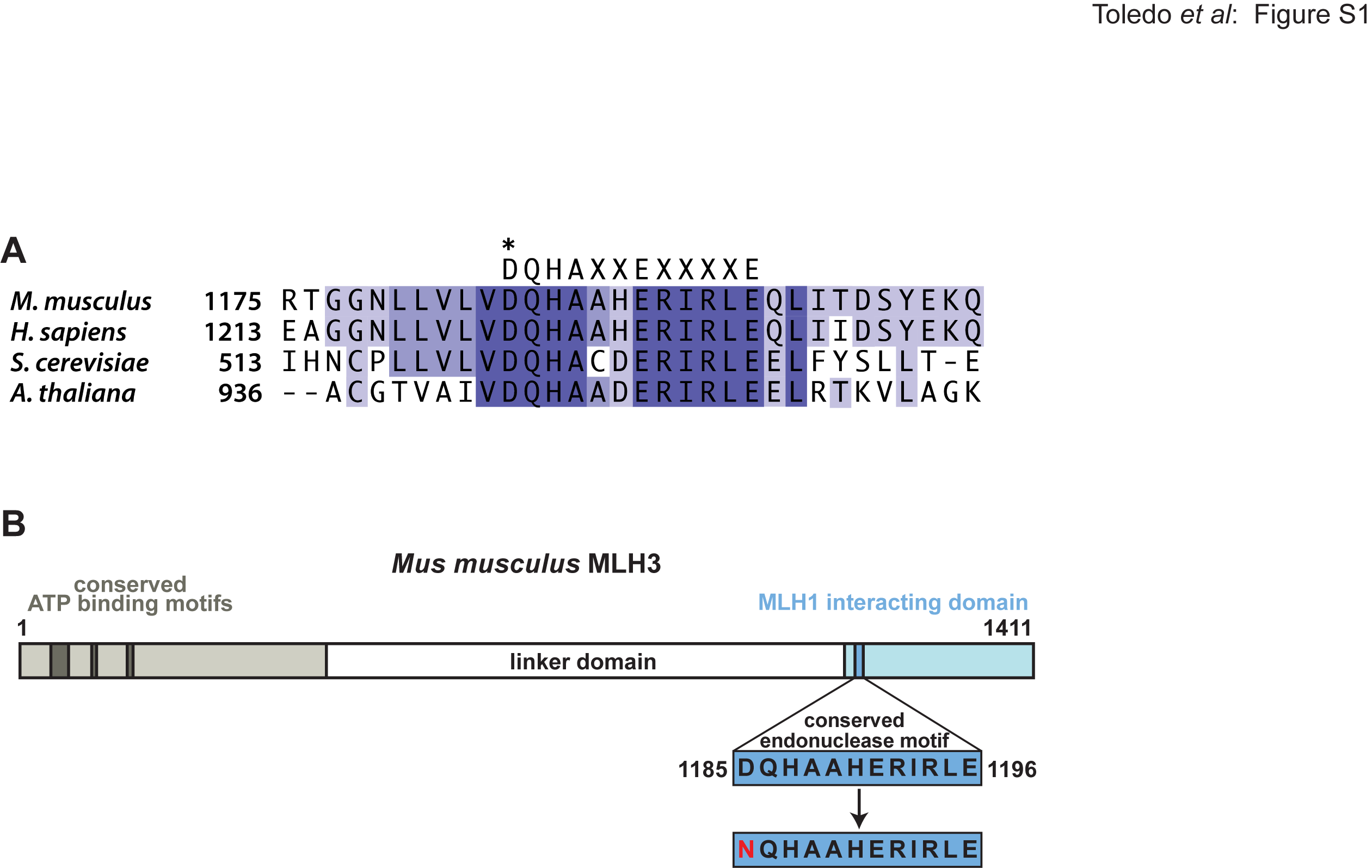

Supplement: S1 Fig — (A) Amino acid protein sequence of the M. musculus, H. sapiens, S. cerevisiae, and A. thaliana MLH3 endonuclease domain, DQHA(X)2E(X)4E, shows the conservation of this domain across these species. Asterisk refers to the conserved aspartic acid (D) that was targeted for a point mutation and converted to asparagine (N) to generate the Mlh3-DN mouse. (B) Mus musculus MLH3 is composed of a 1411 amino acid long sequence that results in an ~158 kDa sized protein (UniProt, 2015). MLH3 contains a globular N-terminal domain (NTD; light gray) and C-terminal domain (CTD; light blue) connected by a flexible linker arm (white). The NTD (light gray) contains ATP binding motifs (dark gray) that are conserved across species. The CTD consists of the MLH1 interacting domain (light blue) and the conserved endonuclease motif (dark blue). The aspartic acid (D) in the conserved endonuclease motif in mouse, DQHAAHERIRLE, was converted to an asparagine (N; red) at amino acid site 1185. (TIF) [file pgen.1008177.s001.tif]

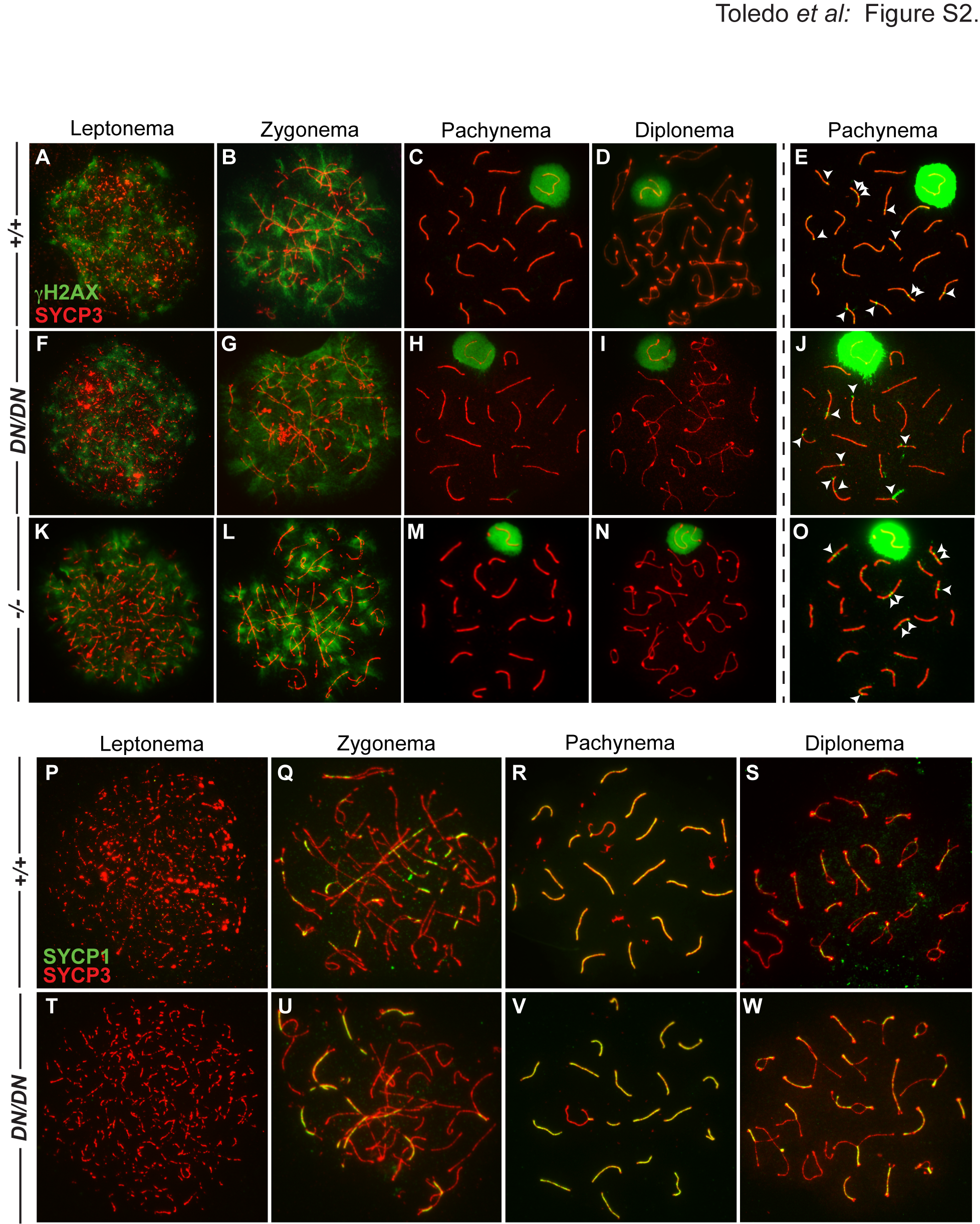

Supplement: S2 Fig — (A-O) Mlh3DN/DN prophase I cells exhibit grossly normal DSB formation and signaling as observed by γH2AX staining (green) on synaptonemal complex protein SYCP3 (red) as compared to WT and Mlh3-/- cells. Images show abundant γH2AX signal in leptonema, following by diminished signal in zygonema, with the absence of signal in pachynema and diplonema, except at the sex body, due to MSCI. (E, J, O) Over exposure of the γH2AX signal results in γH2AX foci or flares on the autosomes in WT, Mlh3DN/DN, as well as Mlh3-/- cells (white arrows), suggesting that this signal may not be representative of true un-repaired DSBs. (P-W) Mlh3DN/DN prophase I cells have normal synapsis as observed by the localization of synaptonemal complex protein SYCP1 (green) and SYCP3 (red) on the chromosomes when compared to WT. SYCP3 forms as short patches along the chromosomes in leptonema, extending into filaments in zygonema along with the appearance of SYCP1, full synapsis with the co-localization of SYCP1 and SYCP3 are observed in pachynema, followed by desynapsis in diplonema with the degradation of SYCP1. (TIF) [file pgen.1008177.s002.tif]

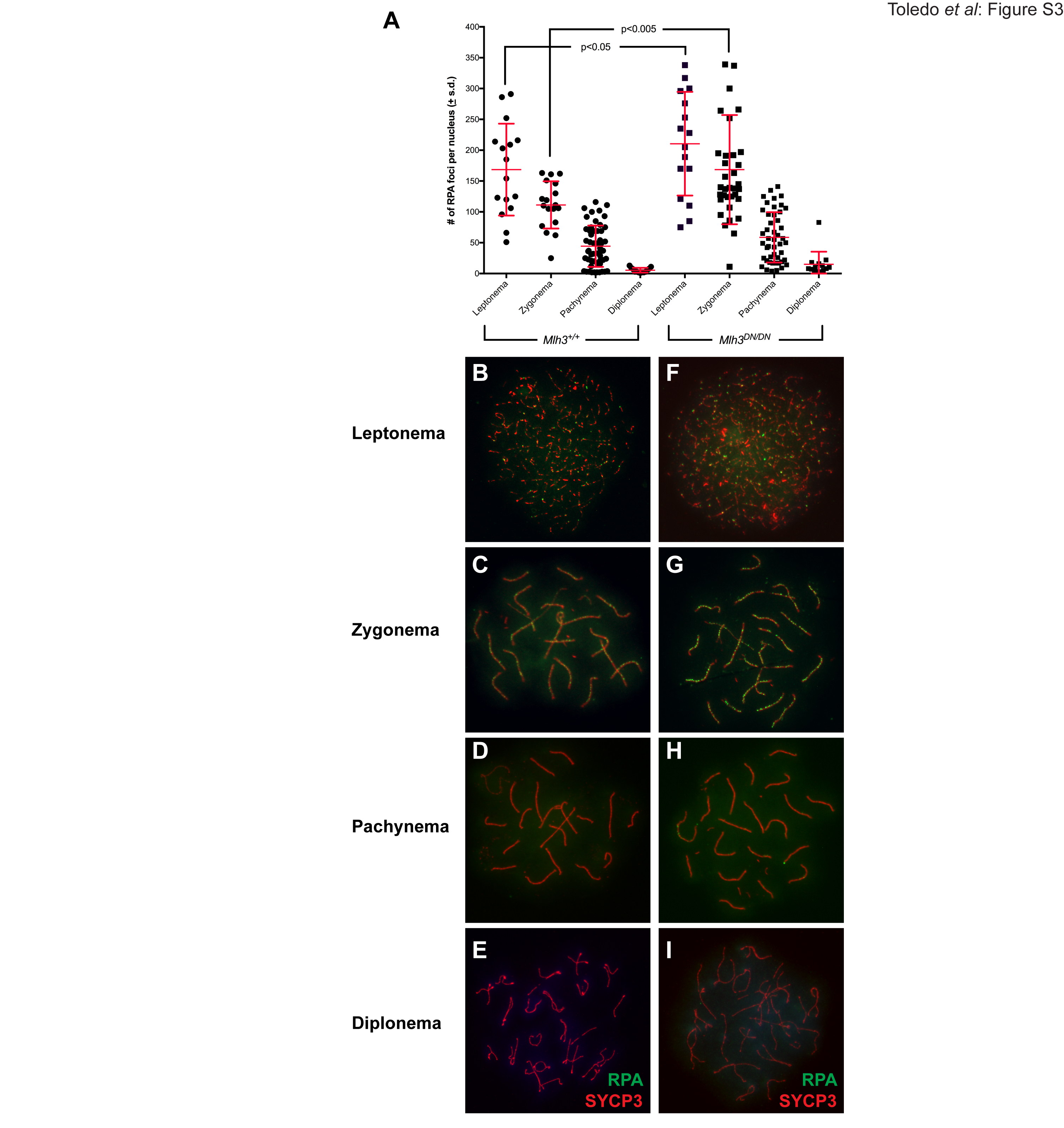

Supplement: S3 Fig — (A) Quantitation of RPA foci in spermatocytes from leptonema through diplonema shows initially elevated RPA numbers, with a progressive decline through prophase I for both WT and Mlh3DN/DN spermatocytes. However, at leptonema and diplonema, the RPA focus counts are significantly elevated in Mlh3DN/DN spermatocytes compared to WT littermate controls (p values given in graph: unpaired t-test with Welch’s correction). Values given are number of foci per nucleus ± s.d. (B-I) Example spread images of RPA staining at different prophase I stages, including leptonema (B,F), zygonema (C,G), pachynema (D,H), and diplonema (E,I) from Mlh3+/+ (B-E) and Mlh3DN/DN (F-I) male mice. Chromosome spreads were stained with antibodies against SYCP3 (red) and RPA (green). (TIF) [file pgen.1008177.s003.tif]

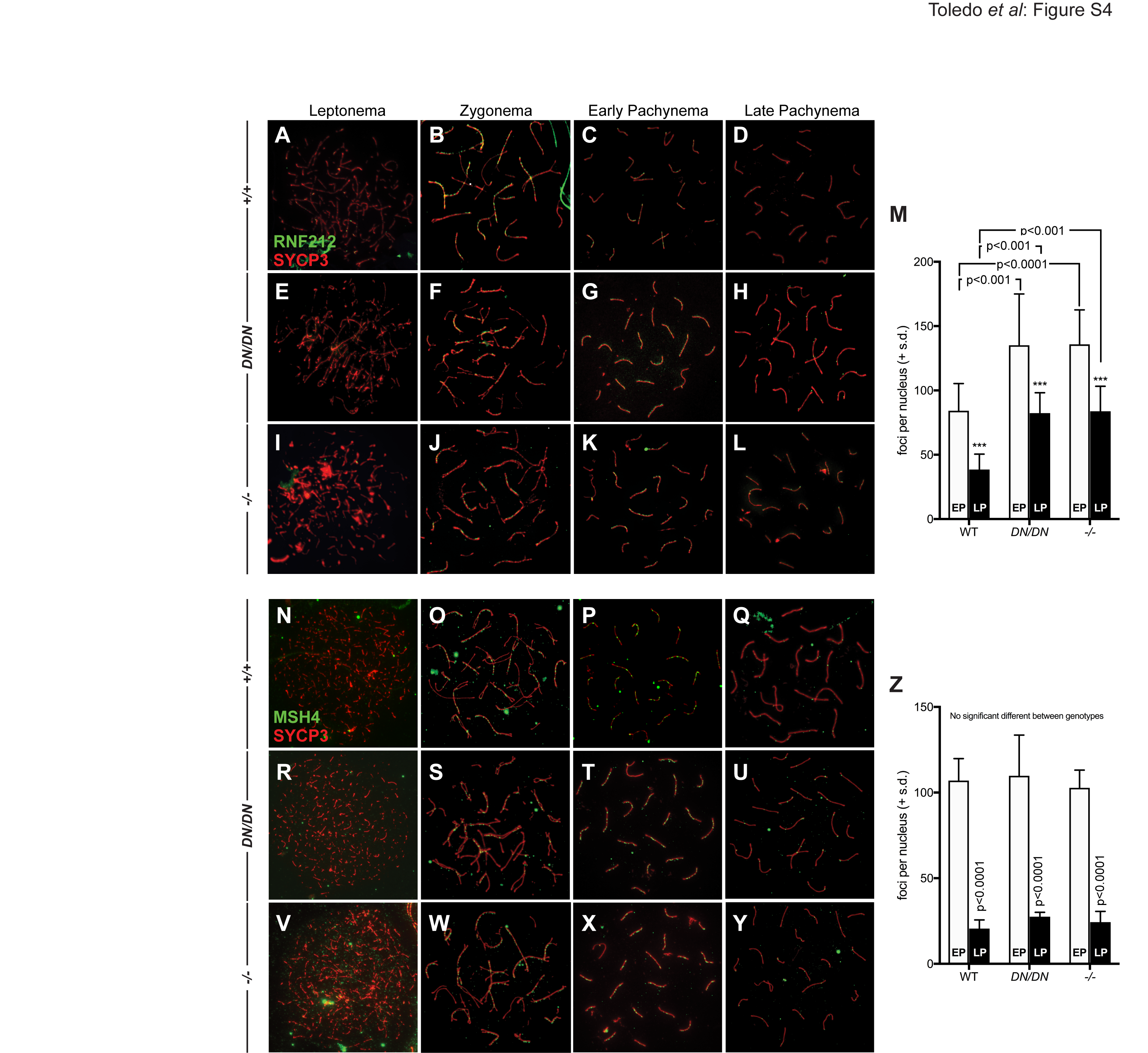

Supplement: S4 Fig — (A-M) RNF212 (green) localization on chromosome cores stained with antibodies against SYCP3 (red) through early prophase I (leptonema, zygonema, early pachynema, and late pachynema). Representative images of spermatocytes from WT (A-D), Mlh3DN/DN (E-H), and Mlh3-/- (I-L) adult males. Panel M shows the quantitation of RNF212 foci in all three genotypes at early (EP) and late pachynema (LP). RNF212 accumulates on chromosome cores at zygonema in high numbers and these foci diminish gradually through pachynema with only one or two foci remaining in late pachynema. (N-Z) MSH4 (green) localization on chromosome cores stained with antibodies against SYCP3 (red) through early prophase I (leptonema, zygonema, early pachynema, and late pachynema). Representative images of spermatocytes from WT (M-P), Mlh3DN/DN (Q-T), and Mlh3-/- (U-X) adult males. Panel Z shows the quantitation of MSH4 foci in all three genotypes at early (EP) and late pachynema (LP). A similar pattern of MSH4 foci accumulation and loss is observed for MSH4 as for RNF212: accumulation of high numbers of foci in zygonema, diminishing to one or two foci per chromosome in late pachynema. In all cases, statistical analysis was performed using unpaired t-test with Welch’s correction (p values provided in graphs), with Bonferroni’s adjustment for multiple comparisons where necessary. For all chromosome imaging and foci counts, at least three mice of each genotype were observed for each staining set. (TIF) [file pgen.1008177.s004.tif]

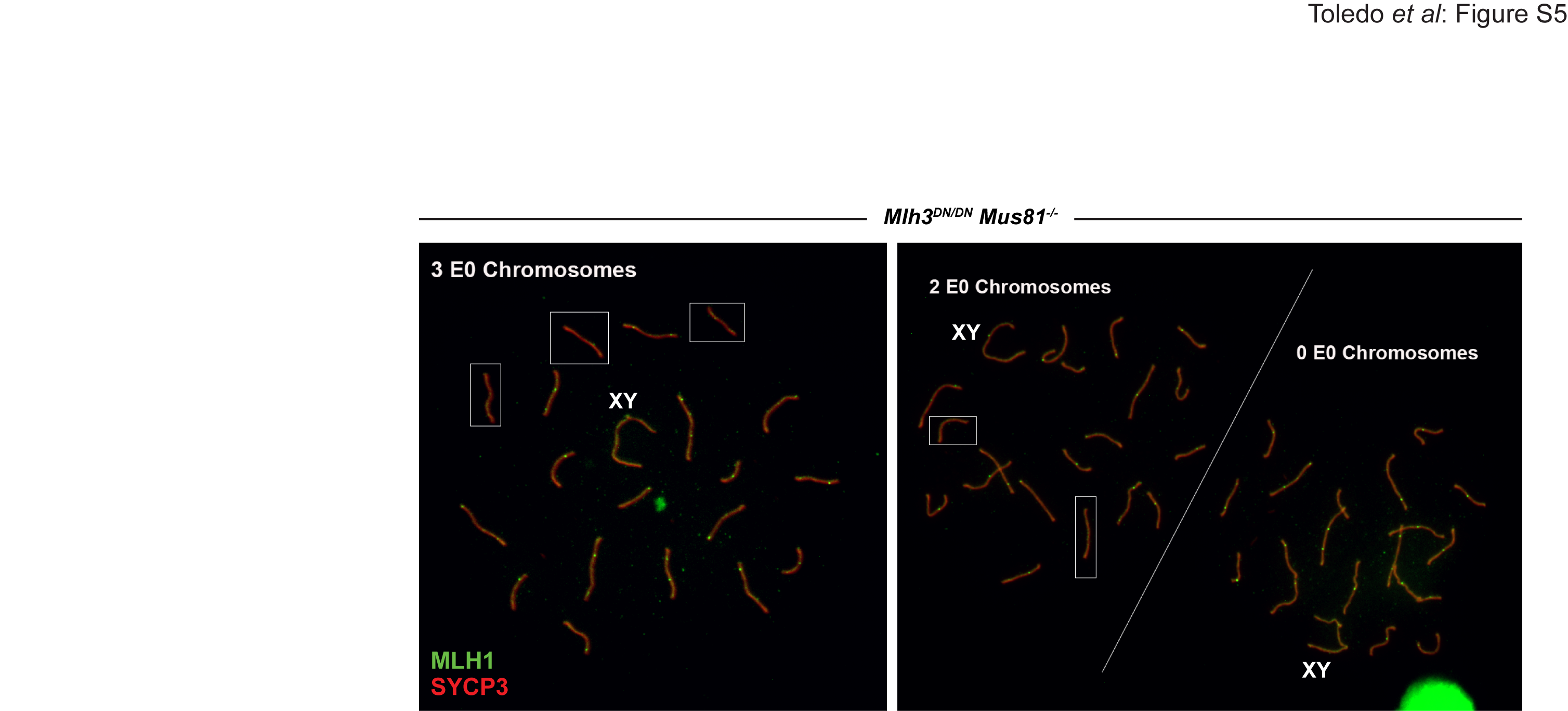

Supplement: S5 Fig — E0 chromosomes are highlighted with a white box. The XY bivalent is indicated (but was not included in the counts because the MLH1 focus is not always visible on the PAR). (TIF) [file pgen.1008177.s005.tif]
